# Concurrent and longitudinal associations between touchscreen use and executive functions at preschool-age

**DOI:** 10.3389/fdpys.2024.1422635

**Published:** 2024-08-14

**Authors:** Alicia Mortimer, Abigail Fiske, Bethany Biggs, Rachael Bedford, Alexandra Hendry, Karla Holmboe

**Affiliations:** 1 Department of Experimental Psychology, University of Oxford, Oxford, United Kingdom; 2 School of Experimental Psychology, Univeristy of Bristol, Bristol, United Kingdom; 3 Department of Biological and Experimental Psychology, Queen Mary University of London, London, United Kingdom

**Keywords:** touchscreen, executive functions, preschool cognitive development, media exposure, active screen use, passive screen use

## Abstract

**Introduction:**

The prevalence of touchscreen devices has recently risen amongst young children. Some evidence suggests that increased touchscreen use may be negatively related to preschool-age children’s executive functions (EFs). However, it has been argued that actively interacting with touchscreen devices (e.g., via creative apps for drawing) could better support EF development compared to passive use (e.g., watching videos). There is a pressing need to understand whether the type of use can explain potential associations between touchscreen use and EF.

**Methods:**

By following up longitudinally on an infant sample, now aged 42-months (*N* = 101), the current study investigates the relative contributions of passive and active touchscreen use, measured concurrently at 42-months and longitudinally from 10-to-42-months, on parent-reported EFs.

**Results:**

A multivariate multiple regression found no significant negative associations between touchscreen use and preschool EF. There was a significant positive association between active touchscreen use at 42-months and the BRIEF-P Flexibility Index.

**Discussion:**

The lack of significant negative associations found is consistent with an earlier study’s findings in the same sample at infancy, suggesting that the moderate levels of early touchscreen use in this sample are not significantly associated with poorer EF, at least up to preschool-age.

## Introduction

Executive functions (EFs) are core cognitive skills needed to control our attention and purposeful behaviors to work toward goals in everyday life (Blair, 2016). EF skills include inhibitory control (IC; deliberately supressing dominant yet inappropriate responses), working memory (WM; actively maintaining important information in mind), and cognitive flexibility (CF; considering simultaneous representations of an object or event and/or flexibly alternating between tasks). EF skills develop rapidly during early childhood and play an important role in social and academic school readiness (Blair et al., 2005; Hendry et al., 2016). EFs rely on the development of the prefrontal cortex (Best and Miller, 2010; Fiske and Holmboe, 2019), which is thought to be particularly susceptible to individual differences in children’s early environments (Hodel, 2018). Several environmental factors, including maternal mood (Power et al., 2021), socioeconomic status (Lawson et al., 2018), as well as traditional screen media (e.g., television; Kostyrka-Allchorne et al., 2017a) have been linked to developmental differences in EF. In recent years there has been an increase in the use of touchscreen devices (e.g., tablet devices and smartphones) amongst young children (Bedford et al., 2016; Bergmann et al., 2022; Hendry et al., 2022). The increased portability of touchscreen devices may make them more easily accessible to young children, potentially increasing the opportunity to impact early EF development (Jusiene et al., 2020; Eric, 2021; Taherian Kalati and Kim, 2022). However, the relatively recent increase in popularity of touchscreen devices amongst young children means that research addressing associations between touchscreen media and early EF development is still limited. The aim of this study was to test the association between duration and type of touchscreen use and EF skills in preschool-age children.

Given the relatively limited research investigating the impact of touchscreen use on early EF skills, it is important to consider the impact of traditional screen media (i.e., television, TV) on young children’s cognitive development. Christakis et al. (2004) found that TV exposure before 3-years was associated with parent-reported attentional problems at 7-years. Similarly, Miller et al. (2007) found that TV viewing was associated with more inattentive/hyperactive behaviors amongst preschoolers, which could have a negative impact on early EF skills considering that maturing attentional control forms the basis of preschool EF development (Garon et al., 2008). Experimental studies investigating the effect of immediate viewing of fantastical screen content, which potentially violates children’s knowledge/expectations of reality, have also shown links to reduced EF proficiency post-television viewing (in comparison to EFs measured pre-television viewing; Rhodes et al., 2020). It may be that such content is difficult for children to incorporate into their pre-existing mental representations, depleting their limited attentional resources needed for successful EF performance (Lee and Lang, 2015; Rhodes et al., 2020). With touchscreen usage becoming increasingly prevalent amongst young children (Kostyrka-Allchorne et al., 2017b), it is important to determine whether touchscreen devices may exacerbate the negative effects of screen media previously found between TV viewing and early EF skills.

Although several studies have found negative associations between touchscreen use and early EF development, the majority of these have employed unitary measures of EF, or combinations of several EF measures to explore general executive functioning (Barr et al., 2010; Lillard and Peterson, 2011; Nathanson et al., 2014; Antrilli and Wang, 2018), despite the different core EF components being identifiable during the preschool period (Garon et al., 2008). IC, WM and CF each have separate developmental trajectories and show differential associations with more complex forms of EF (Friedman et al., 2011; Hendry et al., 2016; Devine et al., 2019; e.g., looking ahead to the attainment of a goal and planning one’s actions accordingly). Investigating different EF skills separately is particularly important considering that across existing research, conclusions regarding the effect of touchscreen use on individual EFs have varied. A longitudinal study by Portugal et al. (2023) found no differences in impulse/self-control between low touchscreen users and high touchscreen users (≥15 min/day) at 42-months, but did find that high users showed reduced performance on lab-based WM and CF tasks. However, these effects became non-significant after controlling for background TV, suggesting the effect found may not be specific to touchscreen devices, but instead related to a child’s broader media environment (Portugal et al., 2023). By contrast, McNeill et al. (2019) found a negative association between preschoolers’ touchscreen use (>30 min/day) and IC measured 12-months later (indexed by a Go/No-Go task), but found no links with WM or CF. Similarly, McHarg et al. (2020) found that parent-report of regular screen media use (including touchscreens) at 4-months predicted poorer performance on a self-regulation task at 14-months, but was unrelated to WM or CF. Lawrence et al. (2020) found similar effects later in childhood, as 32-to-47-month-olds who used touchscreen devices more regularly, and at an earlier age, displayed lower self-regulation as measured by experimental tasks.

One potential factor which could be contributing to these different patterns across studies is the way in which children are using touchscreen devices. Screen use can involve more *“passive”* viewing of screen content that requires little interaction/input from the child (e.g., TV-viewing), or *“active”* use which necessitates interactive and cognitive engagement with a screen-media device (e.g., creative apps for drawing, educational games, etc.; Corkin et al., 2021). Some experts in the field have argued that the increased interactivity of active touchscreen use could better support early cognitive development (Christakis, 2014; Kirkorian, 2018; Corkin et al., 2021). During active touchscreen use, because young children can easily navigate touchscreen devices using swiping and tapping motions with their fingers, they are able to touch and manipulate images and characters on the screen in addition to seeing and hearing them (Li et al., 2018). This multimodal stimulation may make the content more realistic and easier to process for some children (Benski and Fisher, 2013). Therefore, the increased interactivity promoted in active touchscreen use may reduce any negative effects on EFs in comparison to passive video-watching (Subrahmanyam and Greenfield, 2008).

There is some preliminary evidence to support active touchscreen use being more developmentally appropriate for early EF development. Huber et al. (2018) found that children were more likely to perform better on a WM task and a delay of gratification task after playing an educational app than after viewing a fantastical cartoon. Similarly, Li et al. (2018) found that simply watching fantastical video content from a game on an iPad had a negative effect on IC, whilst active interaction with the same game had no effect on IC. In contrast, Helm and McDermott (2022) found that active touchscreen use via playing a cooking game on a tablet, in comparison to having no touchscreen usage and instead completing a similar cooking task with toys, had an immediate negative impact on IC performance. However, the active touchscreen game played still involved some passive video-watching which may have negatively impacted subsequent IC performance. Further investigation is needed to better understand the relative contributions of active and passive touchscreen use on different EF skills. This could help to determine whether there should be different screen media guidelines for young children depending on how touchscreen devices are used.

Age is another important factor to consider when investigating the impact of touchscreen media on early EF skills. Although a study by Lui et al. (2021) found no negative associations between touchscreen use and a composite EF measure at 10-months, Hendry et al. (2022) found a negative association between screen use (including touchscreens) and the same composite EF measure amongst a slightly older sample of young children up to 36-months. Bedford et al. (2016) found that average daily duration touchscreen usage time increased with age from 6-to-36-months. Therefore, it is plausible that touchscreen use needs to build up over the first few years of life to have a detrimental effect on EFs at preschool-age. It is also worth considering that touchscreen usage during toddlerhood and the preschool years may be fundamentally different to usage during infancy. While children start to attend to screen content from infancy, sustained attention increases until mid-childhood (Anderson et al., 1986), suggesting that the opportunity for screen-time to impact development may increase with age as children can attend to screen content for much longer durations of time. Therefore, the possible negative effects of early touchscreen usage may appear later in development during the preschool-years rather than in infancy.

By following up on Lui et al.’s (2021) infant sample, now aged 42-months, the current study aims to investigate how concurrent (42-month) and longitudinal (10-to-42-month average) touchscreen use (predictor) is associated with the development of different EF skills (IC, WM and CF, outcome variables) in early childhood. The current study tested (1) whether we replicate negative effects of touchscreen use on EFs found by previous research now that the sample is preschool-age, potentially due to the accumulation of usage over time (McNeill et al., 2019; Lawrence et al., 2020; McHarg et al., 2020; Hendry et al., 2022; Portugal et al., 2023); and (2) whether the effects were driven by passive or active touchscreen use.

## Materials and methods

### Participants

Participants were 101 42-month-olds (48 boys) from the longitudinal Oxford Early Executive Functions (OEEF) study at the Oxford University BabyLab (see the sample demographics in Table 1). The OEEF study received ethical approval from the University of Oxford (Ref. No. R57972). Parents provided informed consent prior to data collection, which took place from April 2019 to November 2022. The longitudinal design allowed information about the children’s touchscreen media usage to be collected at six timepoints across the first 3.5-years of their lives. Specifically, touchscreen media usage was reported when the sample was 10-, 16-, 24-, 30-, 36-, and 42-months-old. EF was also measured at these six timepoints, but the current study only focused on EF skills measured at 42-months. Participants were recruited from the local hospital, the Oxford University BabyLab volunteer database, and social media, and had to meet at least one of the following criteria to be included in the analysis: (a) born at 36 weeks’ gestation or later or (b) weighing at least 5.5 lbs (2.5 kg) at birth. Three participants were excluded from the final sample of 101 children due to potentially serious health issues (e.g., brain abnormalities, oxygen deprivation at birth). Families received Amazon vouchers, stickers and small toys for participating in the OEEF study.

### Materials

#### Touchscreen use questionnaire (TUQ)

The OEEF team created the 12-item TUQ to measure early touchscreen use via parent-report (the full TUQ can be found in the Supplementary material; Lui et al., 2021). The TUQ was completed by parents (usually the mothers of participants, *N* = 89) online via Qualtrics. At 42-months, 94 parents in the final sample completed the TUQ. The number of participants who completed the TUQ at the previous five timepoints can be seen in Table 2.

Administering the TUQ across these different timepoints allowed both concurrent touchscreen use at 42-months, and average touchscreen use from 10-to-42-months to be measured. To be included in the final sample of 101 participants used for the analyses, all participants needed to provide TUQ data on at least 3/6 of the timepoints, with at least one of these being at 10-,16-, or 24-months, and at least one being at 30-, 36-, or 42-months (only two participants did not meet this criteria).

As part of the TUQ, at each timepoint the duration of participants’ passive and active touchscreen use were individually measured by a single item rated on an ordinal scale. Parents reported their child’s passive touchscreen use via the following item: “In the past week, roughly how long in total did your child spend looking at (but not touching) a touchscreen device? (Not including visits to the BabyLab).” Parents then reported their child’s active touchscreen use via this item: “In the past week, roughly how long in total did your child spend interacting with (tapping or swiping) a touchscreen device? (Not including visits to the BabyLab).” Parents separately rated these passive and active touchscreen duration items on a 7-point Likert scale (1 = <5 min, 2 = 5–20 min, 3 = 20– 60 min, 4 = 1–2 h, 5 = 2–4 h, 6 = 4–6 h, 7 = 7 or more hours). The score of passive touchscreen duration (from the “looking at” item) and the score of active touchscreen duration (from the “interacting with” item) at each timepoint were used in the analyses.

Participants’ average passive and active touchscreen use across the six timepoints were calculated separately. Scores of passive touchscreen duration from 10-to-42-months were averaged to calculate an average passive touchscreen use score across the first 3.5-years of life. Likewise, scores of active touchscreen duration from 10-to-42-months were averaged to calculate an average active touchscreen use score across the first 3.5-years of life. These average passive touchscreen use and average active touchscreen use scores had good internal consistency across timepoints (Cronbach’s α = 0.85; Cronbach’s α = 0.84, respectively).

#### The behavior rating inventory of executive function, preschool version (BRIEF-P)

The BRIEF-P is a parent-reported measure of preschool EFs which has been extensively validated amongst different subgroups of children (Gioia et al., 2000; Bausela Herreras, 2019; e.g., across different cultures and clinical samples). Parents rated how frequently their child had problems with different behaviors during the past 6 months on a 3-point scale (Never, Sometimes, Often). It consists of 63 items in five non-overlapping scales which form three overlapping summary indexes: the Inhibitory Self-Control Index (ISCI), the Flexibility Index (FI), and the Emergent Metacognition Index (EMI). The ISCI is composed of the Inhibit scale (item example: “The child is fidgety, restless or squirmy”) and Emotional Control scale (item example: “The child overreacts to small problems”). The FI is also composed of the Emotional Control scale, as well as the Shift scale (item example: “The child has trouble changing activities”). The EMI is composed of the Working Memory scale (item example: “The child has trouble with activities or tasks that have more than one step) and Plan/Organize scale (item example: “The child does not complete tasks after given directions”). The ISCI, FI and EMI indexes were used as measures of the three EF domains (IC, CF, and WM) within the sample. At 42-months, 101 parents completed the BRIEF-P. Because a higher BRIEF-P score means lower EF skills, for ease of interpretation scores were reversed (by subtracting from 100) so that higher BRIEF-P scores represented stronger EF skills. All of the summary indexes had very good internal consistency (ISCI Cronbach’s α = 0.91, FI Cronbach’s α = 0.87, EMI Cronbach’s α = 0.90).

### Sociodemographic questionnaire

The OEEF team created a questionnaire to collect demographic information from the sample. Parents reported their child’s age and sex, who their primary caregiver was, and information about their household (number of rooms, total family annual income, number of adults in the household). Mothers and fathers separately reported their marital status, number of years in education, and occupation. From this questionnaire, child’s sex and mother’s years in education were used in the current study’s analyses. Child’s sex was controlled for because there is behavioral evidence of sex differences in early EFs (Wiebe et al., 2008). Mother’s years in education (a common proxy for socioeconomic status (SES)) was controlled for because previous research has found that children from lower socioeconomic contexts are more negatively affected by screen use, and children from higher socioeconomic contexts also tend to have stronger EF skills (Bernier et al., 2010; Denham et al., 2015; Ribner et al., 2017). Only 67 participants provided information about their father’s years in education, therefore mother’s years in education was used as a proxy of SES.

### Procedure

As part of the longitudinal OEEF study, data was collected from the sample at 10-, 16-, 24-, 30-, 36-, and 42-months. Participants visited the Oxford University BabyLab at 10-, 16-, and 42-months. During these visits, participants completed an EF task battery and parents completed the TUQ, as well as the BRIEF-P at the 42-month timepoint. Within 2 weeks prior to their 42-month visit, parents also completed the sociodemographic questionnaire. This questionnaire was also completed 2 weeks prior to their 10-month visit, therefore if any participants did not complete the questionnaire at 42-months, the sociodemographic information provided at 10-months was included instead.

At the 24-month timepoint, due to the COVID-19 pandemic, very few participants were able to visit the Oxford University BabyLab after the UK government’s lockdown restrictions were implemented in March 2020. Therefore, the majority of participants completed the TUQ remotely at the 24-month timepoint, and all participants completed the TUQ remotely at the 30- and 36-month timepoints.

### Statistical analysis

IBM SPSS Statistics Version 29.0.2 was used for statistical analysis. The Kolmogorov-Smirnov test, histograms, and normal Q-Q plots showed that all variables violated the assumption of normality, apart from the BRIEF-P EMI index (see Supplementary material). Therefore, Spearman’s Rank correlation coefficient, which is robust against skewed data, assessed bivariate associations between touchscreen use (both passive/active and average/concurrent use) and EF skills (as measured by the BRIEF-P’s Inhibitory Self-Control, Flexibility, and Emergent Metacognition indexes; Bishara and Hittner, 2012). The Benjamini-Hochberg procedure was used to correct the alpha level of 0.05 for the false discovery rate (12 family-wise comparisons, see Supplementary material).

To assess the effects of touchscreen use beyond sociodemographic variables, a multivariate multiple regression was performed to integrate the different variables into one model whilst also adjusting the significance test for the multiple dependent variables. Maternal education (a common proxy for socioeconomic status) and child’s sex were entered into the model as independent variables known to influence early EF development (see the “Sociodemographic Questionnaire” section in the Methodology).

Before conducting the multivariate multiple regression, further preliminary analyses (i.e., Cook’s distance, tolerance and variation inflation factor (VIF) statistics, plots of standardized and predicted residuals) were run to check for violations of normality, linearity, multicollinearity, and homoscedasticity (see the Supplementary material). As some of the independent variables were formed from some of the same TUQ items (i.e., the average touchscreen use scales were partly made up of the 42-month touchscreen use scales), Spearman’s correlations were run between all the independent variables to check for the assumption of no multicollinearity (see Supplementary material; Hinkle et al., 2003). None of the variables had a correlation coefficient higher than 0.8, and all other assumptions were met, so the multivariate multiple regression was performed.

## Results

### Descriptive statistics

Descriptive statistics for the sample’s passive and active touchscreen use at each of the six timepoints from 10-to-42-months of age can be seen in Table 3, showing that usage gradually increased amongst the sample over the first 3.5-years of life. Descriptive statistics for the BRIEF-P indexes at 42-months can be seen in Table 4.

### Correlations between touchscreen use and executive functions

There was a significant positive correlation between 42-month active touchscreen use and scores on the BRIEF-P Flexibility Index (*r*_s_ = 0.27, *p* = 0.01), but this did not survive correction for multiple comparisons (Benjamini-Hochberg adjusted *p* = 0.08). No other significant correlations were found between touchscreen use and executive functions as measured by the BRIEF-P. See Table 5 for the full correlation table.

### Additional analyses

Although touchscreen usage has typically been measured in previous research by a single rating of the duration spent on touchscreen devices in a specific time period (Cheung et al., 2017; McHarg et al., 2020; Corkin et al., 2021; Bergmann et al., 2022; Portugal et al., 2023; e.g., in a week), in addition to weekly duration of touchscreen use, the current sample’s *frequency* of touchscreen use was also measured. Parents separately rated how frequently their child completed different actions on a touchscreen (e.g., how often their child would ‘do drawings or scribbles’ on a touchscreen device). Each action was then classified as either passive or active, allowing passive and active touchscreen use to be further differentiated from one another beyond just duration of touchscreen use. The change in frequency of touchscreen use from 10-to-42-months was very similar to the change in duration of touchscreen use (gradually increasing over time; see the Supplementary material). A correlational analysis between frequency of touchscreen use and the EF skills (as measured by the BRIEF-P) was also run to see if this differed from the correlational analysis with duration of touchscreen use presented above. No significant correlations were found between frequency of touchscreen use and EF skills (see Table 6 for the full correlation table). The Supplementary material includes further discussion of this additional correlational analysis.

### Multivariate multiple regression

The results of the multivariate multiple regression investigating the associations between different types of touchscreen use and each EF skill are reported in Table 7. When child’s sex and years of maternal education were accounted for, no significant negative associations between passive or active touchscreen use and parent-reported preschool EFs were found, whether this was concurrent use at 42-months or average use from 10-to-42-months. However, a positive association was found between concurrent active touchscreen use at 42-months and Flexibility Index scores, such that higher active touchscreen use was associated with better Flexibility Index scores (*p* = 0.03, Wilks’ Lambda = 0.081, partial *η*^2^ = 0.05). The 42-month active touchscreen use model as a whole did not significantly explain EF variation (in all three EF outcome measures), *F*_(3,91)_ = 2.681, *p* = 0.052; partial *η*^2^ = 0.08. None of the other predictors significantly explained variation in any of the EF outcome measures.

## Discussion

In a follow-up of the Lui et al. (2021) infant sample, the current study aimed to test the association between touchscreen use and executive function skills at preschool-age. After controlling for demographic variables (child sex and maternal education), no negative associations were found between touchscreen use and preschool EFs. This is consistent with Lui et al.’s (2021) earlier findings in the same sample, suggesting that the moderate levels of early touchscreen use observed in this sample are not significantly associated with poorer EF skills. One positive association was found between active touchscreen use at 42-months and the BRIEF-P Flexibility Index. This positive association is at least partially consistent with Lui et al.’s (2021) finding of a positive association between overall touchscreen use (combining active and passive touchscreen use) and a parent-reported composite EF score in the same sample at 10-months. However, it is important to note that Lui et al. (2021) combined duration and frequency of touchscreen usage into a single measure of touchscreen use, whereas the current study only found an association between *duration* of active touchscreen use and cognitive flexibility (see Supplementary material for the full correlational analysis between *frequency* of touchscreen use and EFs at 42-months). While touchscreen use is most commonly measured and defined in terms of duration, there is a need for future research to consider how duration vs. frequency of use may differentially impact early EF development.

The current study’s findings are broadly consistent with previous reports of active touchscreen usage being less detrimental to EF abilities than passive usage (Huber et al., 2018; Li et al., 2018; Hu et al., 2020; Bustamante et al., 2023). If replicated in other samples, what could explain the positive association between active touchscreen use and CF found in the present study? It has been hypothesized that CF may be exercised and practiced by switching between engaging with a screen-based activity and other activities (e.g., interacting with a parent), or quickly switching between different screen-based activities. This task-switching at a young age may enhance the ability to adapt and switch successfully from one task to another by minimizing task-switching costs (Alzahabi and Becker, 2013). Some research has shown that practicing one’s ability to switch between actions, objectives and rules when interacting with screen media (i.e., engaging in more than one media or non-media activity simultaneously) can train and improve CF in other contexts (Alzahabi and Becker, 2013; Murphy and Shin, 2022).

However, the lack of direct EF assessment is a limitation of the current study, and the positive association found between active touchscreen use and CF needs to be replicated using experimental measures of CF in addition to parental report. Portugal et al. (2023) actually found that 42-month-old children with high levels of touchscreen use had poorer performance on a composite experimental measure of CF and WM. It may be the case that parental reports vs. experimental tasks assess different aspects of EF (Toplak et al., 2013). Although experimental measures of EF were also used in the OEEF study, the current paper focused on parent-reported EF because parental report tends to measure more ecologically valid aspects of EF (such as pursuing everyday goals), whereas EF tasks relate to accuracy of test performance and processing efficiency (Toplak et al., 2013). It remains an important aim for future research to tease apart the association between touchscreen use and experimental tasks vs. parent-reported EF.

In addition to the Shift scale, the BRIEF-P Flexibility Index is made up of the Emotional Control scale. Although previous research has found increased overall touchscreen use (combining passive and active touchscreen use) to be associated with lower self-regulation (Lawrence et al., 2020), the positive association found in the present study could suggest that moderate amounts of active touchscreen use may not show the same negative associations. In line with this, no associations were found between touchscreen usage and the BRIEF-P Inhibitory Self-Control Index (which consists of the Emotional control scale and Inhibit scale). Additionally, no associations were found between touchscreen usage and the Emergent. Metacognition Index (which consists of the Working Memory scale and Plan/Organize scale). WM and IC seem to influence and support each other over the preschool period, with performance on WM and inhibition tasks correlating with one another (Senn et al., 2004). It has been hypothesized that CF improves particularly rapidly during the preschool period, and that CF abilities are theoretically built on WM and IC which may have already undergone rapid development earlier in life (Scionti and Marzocchi, 2021). Therefore, CF may be more sensitive to external influences of EF development, such as touchscreen usage, during the preschool period in comparison to IC and WM.

It is also important to consider how touchscreen use is related to a young child’s broader media environment, which often involves a mixture of TV, tablets, smartphones, and video game consoles. The negative association found by Portugal et al. (2023) between touchscreen use and CF/WM was no longer significant once background TV was controlled for. Although a recent study by Brauchli et al. (2024) found that general screen time (including both TV and touchscreen use) did not influence 12-to-36-month-olds’ effortful control (a construct related to EF), many other contextual and content-related screen media factors in a child’s environment were not considered. For example, the impact of background TV likely depends on many factors, including the number of TVs in a home and how many hours a child spends at home. Unfortunately, no data about the OEEF sample’s broader media environment beyond touchscreen devices was collected. Additionally, passive and active touchscreen use being measured only by a single item each is a clear limitation of the current study by potentially oversimplifying children’s diverse use of screen media. Therefore, the use of more nuanced and objective measures of children’s duration, content, and usage-type of various media platforms could produce a more comprehensive picture of children’s media environment in future studies. This will allow for a better understanding of how different ways in which screen media are used can influence the early development of not only EFs, but also other cognitive domains (such as language). For example, Neumann and Neumann (2014) found that touchscreen usage was positively associated with emergent literacy skills in preschoolers, but that this association was dependent on many important factors beyond just tablet use time, such as quality of content.

### Strengths and limitations

To date, no study has separately investigated the longitudinal and concurrent effects of passive and active touchscreen use on EF in such a large sample of preschoolers. By collecting data on a wide range of active touchscreen usages, future research can more specifically pinpoint where any positive impacts of active touchscreen use may lie.

The longitudinal design of the current study allowed us to consider children’s touchscreen use across the first 3.5-years of life, as well as concurrent covariation. Children’s capabilities and developmental needs undergo significant changes during the preschool period, and children may be more vulnerable to the effects of environmental influences such as screen media usage at different ages (Zelazo and Carlson, 2012; Horowitz-Kraus et al., 2023). This is why the preschool period is arguably the optimal time to investigate whether screen media influences essential EF and EF-related skills (e.g., academic and socio-emotional skills; Conway and Stifter, 2012). Importantly, however, measuring touchscreen use *across* the first 3.5-years of life guards against temporary fluctuations at one specific age. We did not find any associations between average passive or active touchscreen use from 10-to-42-months and preschool EFs. Although not corroborating the potential beneficial effect of active usage on cognitive flexibility, this result supports the conclusion of Lui et al. (2021) that there is no obvious negative impact of touchscreen use on EFs (at least within a relatively high-SES sample), extending this finding up to 42-months of age.

Several limitations of the current study should be considered. Firstly, we cannot determine the causal direction of the association found between 42-month active touchscreen use and the BRIEF-P Flexibility Index. It may be that children who already have stronger flexibility skills could be more motivated to actively engage with touchscreen devices. Their stronger flexibility skills could enable them to more successfully process information presented both on and off screens, as well as enable them to apply any flexibility-related skills taught to them via touchscreens in other contexts which do not involve screens. This highlights the importance of future research considering a range of other pre-existing differences related to EF which could drive differences in touchscreen usage, or mediate the relationship between touchscreen use and EFs.

In relation to considering other covariates, although maternal years in education was controlled for as a proxy for SE background, the sample was broadly from a high-SE context with moderate-to-low touchscreen use levels. This lack of variation in touchscreen use levels meant that longitudinal touchscreen trajectories could not be estimated to allow for trajectory-based comparisons. Previous research has found that children from lower SE contexts are typically exposed to longer durations of screen-based media (Barr et al., 2010; Kostyrka-Allchorne et al., 2017a). Hence, the current study’s sample characteristics may have resulted in the (mainly) null results, and the potential negative impacts of excessive touchscreen use cannot be ruled out. Studying children with excessive screen media use is particularly important as this group has been found to be at an elevated risk for emotional and behavioral problems and low self-regulation skills (Lawrence et al., 2020; Gueron-Sela et al., 2023). Future research should test the potential cumulative impact of touchscreen use using growth curve and growth mixture modeling in a larger cohort of children with more varied levels of touchscreen use than the current sample. This would allow for trajectory- and class-based comparisons to better understand the impact of excessive touchscreen use on early cognitive development.

## Conclusion

Using data from the OEEF study (a large longitudinal study investigating early EF development), the present study investigated the potential associations between touchscreen use and the development of preschool EFs. The relative contributions of concurrent and longitudinal passive and active touchscreen use on preschool EF development were tested. Contrary to some previous findings, touchscreen use was not negatively associated with EFs, and active touchscreen use at 42-months was positively associated with parent-reported scores on the BRIEF-P Flexibility Index. Distinguishing between the effects of different types of touchscreen use on EF development, and how these relate to a child’s broader media environment, will be key for policymakers and early years practitioners to create more nuanced, evidence-based guidelines.

## Supplementary Material

The Supplementary Material for this article can be found online at: https://www.frontiersin.org/articles/10.3389/fdpys.2024.1422635/full#supplementary-material

Supplementary material

## Figures and Tables

**Table 1:** Sample’s demographic characteristics.

Characteristic	*N*	Mean	*SD*	Min	Max
Child’s age (in months)*	101	42.06	0.45	41.09	43.78
Mother’s years of education**	100	18.20	3.24	8	30
	*N*	%			
Child’s Sex					
Male	48	47.52			
Female	53	52.48			
Child’s Ethnicity					
White British	73	72.28			
White and Mexican	1	0.99			
White and Black Caribbean	1	0.99			
White and Black African	1	0.99			
White and Asian	4	3.96			
White and Arabic	1	0.99			
Other White	15	14.85			
Other Mixed	1	0.99			
Asian	1	0.99			
Prefer not to say	1	0.99			
Unanswered	2	1.98			

* Age at which the sample’s preschool EFs were measured.

** 1 missing response.

**Table 2:** Frequency of TUQ responses at each timepoint.

Timepoint	*N*	Mean Age*	Age SD	Min Age	Max Age
10-months	100	10.07	0.28	9.67	11.45
16-months	97	16.22	0.37	15.63	18.29
24-months	85	24.38	0.37	23.75	25.79
30-months	82	30.48	0.60	29.80	32.63
36-months	86	36.45	0.56	35.39	37.99
42-months	94	42.07	0.41	41.12	43.29

* Mean age (in months) of the child when the TUQ was completed at each timepoint.

**Table 3:** Descriptive statistics of passive and active touchscreen use at each timepoint.

	10-months	16-months	24-months	30-months	36-months	42-months	Average (10–42 months)
Passive touchscreen use*							
*Mean*	1.86	2.39	3.07	3.36	3.21	3.37	2.84
*SD*	1.14	1.45	1.58	1.96	1.76	1.83	1.23
Active touchscreen use**							
*Mean*	1.26	1.84	2.33	2.66	2.28	2.79	2.22
*SD*	0.69	1.25	1.59	1.79	1.77	1.58	1.06

This table only includes participants who met the criteria of completing the TUQ on at least 3/6 of the timepoints, with at least one of these being at 10-,16-, or 24-months, and at least one being at 30-, 36-, or 42-months (N = 101).

* Passive touchscreen use = the duration a child spent looking at (but not touching) a touchscreen device in the past week (rated on a 7-point Likert scale from “<5 min” to “7 or more hours”).

** Active touchscreen use = the duration a child spent interacting with (tapping or swiping) a touchscreen device in the past week (rated on a 7-point Likert scale from “<5 min” to “7 or more hours”).

ISCI, Inhibitory Self-Control Index; FI, Flexibility Index; EMI, Emergent Metacognition Index.

**Table 4:** Descriptive statistics of the reversed BRIEF-P indexes.

EF Index	*N*	Mean	*SD*	Min	Max
Inhibitory self-control	101	48.56	9.14	13	64
Flexibility	101	48.38	8.95	13	65
Emergent metacognition	101	47.93	10.56	24	66

**Table 5:** Correlations between touchscreen use and EF skills as measured by the BRIEF-P.

Variables	42-month Passive Touchscreen Use	42-month Active Touchscreen Use	Average Passive Touchscreen Use	Average Active Touchscreen Use
ISCI scores	0.04	0.13	0.06	0.08
FI scores	−0.08	0.27*	−0.07	0.14
EMI scores	0.10	0.04	0.14	0.07

* p < 0.05, two-tailed.

ISCI, Inhibitory Self-Control Index; FI, Flexibility Index; EMI, Emergent Metacognition Index.

**Table 6:** Correlations between touchscreen use frequency and the BRIEF-P indexes.

Variables	Frequency of 42-month Passive Use	Frequency of 42-month Active Use	Frequency of Average Passive Use	Frequency of Average Active Use
ISCI scores	−0.11	0.03	−0.14	0.03
FI scores	−0.06	0.07	−0.13	0.10
EMI scores	−0.09	−0.004	−0.04	−0.03

* p < 0.05, two-tailed.

ISCI, Inhibitory Self-Control Index; FI, Flexibility Index; EMI, Emergent Metacognition Index.

**Table 7:** Multivariate multiple regression for the variables predicting each EF domain.

Predictors	B	SE	*p* value	95% CI
Inhibitory Self-Control Index				
Child sex	−1.48	1.96	0.45	(−5.37, 2.41)
Maternal education	−0.32	0.32	0.32	(−0.96,0.31)
42-month passive touchscreen use	−0.19	0.86	0.82	(−1.90, 1.51)
42-month active touchscreen use	0.85	0.94	0.37	(−1.01, 2.71)
Average passive touchscreen use	0.58	1.29	0.65	(−1.97, 3.15)
Average active touchscreen use	−0.44	1.59	0.79	(−3.60, 2.73)
Flexibility Index				
Child sex	−1.42	1.83	0.44	(−5.05, 2.20)
Maternal education	−0.53	0.30	0.08	(−1.13,0.06)
42-month passive touchscreen use	0.04	0.80	0.96	(−1.55, 1.63)
42-month active touchscreen use	1.99	0.87	0.03*	(0.25, 3.72)
Average passive touchscreen use	−0.71	1.21	0.56	(−3.10, 1.69)
Average active touchscreen use	−1.10	1.48	0.46	(−4.04, 1.85)
Emergent Metacognition Index				
Child sex	0.21	2.27	0.93	(−4.31, 4.72)
Maternal education	0.15	0.37	0.68	(−0.59.89)
42-month passive touchscreen use	−0.23	1.00	0.82	(−2.21, 1.74)
42-month active touchscreen use	−0.26	1.09	0.81	(−2.42, 1.90)
Average passive touchscreen use	0.73	1.50	0.63	(−2.25, 3.71)
Average active touchscreen use	0.71	1.85	0.70	(−2.96, 4.38)

* p < 0.05, two-tailed.

## Data Availability

The raw data supporting the conclusions of this article will be made available by the authors, without undue reservation.

## References

[R1] Alzahabi R, Becker MW (2013). The association between media multitasking, task-switching, and dual-task performance. J Exp Psychol.

[R2] Anderson DR, Lorch EP, Field DE, Collins A, Nathan JG (1986). Television viewing at home: age trends in visual attention and time with TV. Child Dev.

[R3] Antrilli NK, Wang SH (2018). Toddlers on touchscreens: immediate effects of gaming and physical activity on cognitive flexibility of 2.5-year-olds in the US. J Child Media.

[R4] Barr R, Lauricella A, Zack E, Calvert SL (2010). Infant and early childhood exposure to adult-directed and child-directed television programming: relations with cognitive skills at age four. Merrill-Palmer Quart.

[R5] Bausela Herreras E (2019). BRIEF-P: validation study in children in early childhood with neurodevelopmental disorders. Sage Open.

[R6] Bedford R, Saez de Urabain IR, Cheung CH, Karmiloff-Smith A, Smith TJ (2016). Toddlers’ fine motor milestone achievement is associated with early touchscreen scrolling. Front Psychol.

[R7] Benski T, Fisher E, Boyns D, Loprieno D (2013). Feeling Through Presence: Toward a Theory of Interactions Rituals and Parasociality of Online Social Worlds.

[R8] Bergmann C, Dimitrova N, Alaslani K, Almohammadi A, Alroqi H, Aussems S (2022). Young children’s screen time during the first COVID-19 lockdown in 12 countries. Sci Rep.

[R9] Bernier A, Carlson SM, Whipple N (2010). From external regulation to self-regulation: early parenting precursors of young children’s executive functioning. Child Dev.

[R10] Best JR, Miller H (2010). A developmental perspective on executive function. Child Dev.

[R11] Bishara AJ, Hittner JB (2012). Testing the significance of a correlation with nonnormal data: comparison of Pearson, Spearman, transformation, and resampling approaches. Psychol Methods.

[R12] Blair C (2016). Executive function and early childhood education. Curr Opin Behav Sci.

[R13] Blair C, Zelazo D, Greenberg MT (2005). The measurement of executive function in early childhood. Dev Neuropsychol.

[R14] Brauchli V, Edelsbrunner P, Castro RP, Barr R, von Wyl A, Lannen P, Sticca F (2024). Screen time vs. scream time: developmental interrelations between young children’s screen time, negative affect, and effortful control. Comput Human Behav.

[R15] Bustamante JC, Fernández-Castilla B, Alcaraz-Iborra M (2023). Relation between executive functions and screen time exposure in under 6 year-olds: a meta-analysis. Comput Human Behav.

[R16] Cheung CH, Bedford R, De Urabain Saez, Karmiloff-Smith A, Smith TJ (2017). Daily touchscreen use in infants and toddlers is associated with reduced sleep and delayed sleep onset. Sci Rep.

[R17] Christakis DA (2014). Interactive media use at younger than the age of 2 years: time to rethink the American Academy of Pediatrics guideline?. JAMA Pediat.

[R18] Christakis DA, Zimmerman FJ, DiGiuseppe DL, McCarty CA (2004). Early television exposure and subsequent attentional problems in children. Pediatrics.

[R19] Conway A, Stifter CA (2012). Longitudinal antecedents of executive function in preschoolers. Child Dev.

[R20] Corkin MT, Peterson ER, Henderson AM, Waldie KE, Reese E, Morton SM (2021). Preschool screen media exposure, executive functions and symptoms of inattention/hyperactivity. J Appl Dev Psychol.

[R21] Denham SA, Bassett HH, Sirotkin YS, Brown C, Morris CS (2015). “No-ooo peeking”: Preschoolers’ executive control, social competence, and classroom adjustment. J Res Childh Educ.

[R22] Devine RT, Ribner A, Hughes C (2019). Measuring and predicting individual differences in executive functions at 14 months: a longitudinal study. Child Dev.

[R23] Eric O (2021). The negative effects of new screens on the cognitive functions of young children require new recommendations. Ital J Pediatr.

[R24] Fiske A, Holmboe K (2019). Neural substrates of early executive function development. Dev Rev.

[R25] Friedman NP, Miyake A, Robinson JL, Hewitt JK (2011). Developmental trajectories in toddlers’ self-restraint predict individual differences in executive functions 14 years later: a behavioral genetic analysis. Dev Psychol.

[R26] Garon N, Bryson SE, Smith IM (2008). Executive function in preschoolers: a review using an integrative framework. Psychol Bull.

[R27] Gioia GA, Isquith K, Guy SC, Kenworthy L (2000). Test review behavior rating inventory of executive function. Child Neuropsychol.

[R28] Gueron-Sela N, Shalev I, Gordon-Hacker A, Egotubov A, Barr R (2023). Screen media exposure and behavioral adjustment in early childhood during and after COVID-19 home lockdown periods. Comput Human Behav.

[R29] Helm AF, McDermott JM (2022). Impact of tablet use on young children’s inhibitory control and error monitoring. J Exp Child Psychol.

[R30] Hendry A, Greenhalgh I, Bailey R, Fiske A, Dvergsdal H, Holmboe K (2022). Development of directed global inhibition, competitive inhibition and behavioural inhibition during the transition between infancy and toddlerhood. Dev Sci.

[R31] Hendry A, Jones EJ, Charman T (2016). Executive function in the first three years of life: Precursors, predictors and patterns. Dev Rev.

[R32] Hinkle DE, Wiersma W, Jurs SG (2003). Applied Statistics for the Behavioral Sciences.

[R33] Hodel AS (2018). Rapid infant prefrontal cortex development and sensitivity to early environmental experience. Dev Rev.

[R34] Horowitz-Kraus T, Fotang J, Niv L, Apter A, Hutton J, Farah R (2023). Executive functions abilities in preschool-age children are negatively related to parental EF, screen-time and positively related to home literacy environment: an EEG study. Child Neuropsychol.

[R35] Hu BY, Johnson GK, Teo T, Wu Z (2020). Relationship between screen time and Chinese children’s cognitive and social development. J Res Childh Educ.

[R36] Huber B, Yeates M, Meyer D, Fleckhammer L, Kaufman J (2018). The effects of screen media content on young children’s executive functioning. J Exp Child Psychol.

[R37] Jusiene R, Rakickiene L, Breidokiene R, Laurinaityte I (2020). Executive function and screen-based media use in preschool children. Infant Child Dev.

[R38] Kirkorian HL (2018). When and how do interactive digital media help children connect what they see on and off the screen?. Child Dev Perspect.

[R39] Kostyrka-Allchorne K, Cooper NR, Simpson A (2017a). The relationship between television exposure and children’s cognition and behaviour: a systematic review. Dev Rev.

[R40] Kostyrka-Allchorne K, Cooper NR, Simpson A (2017b). Touchscreen generation: children’s current media use, parental supervision methods and attitudes towards contemporary media. Acta Paediatr.

[R41] Lawrence AC, Narayan MS, Choe DE (2020). Association of young children’s use of mobile devices with their self-regulation. JAMA Pediatr.

[R42] Lawson GM, Hook CJ, Farah MJ (2018). A meta-analysis of the relationship between socioeconomic status and executive function performance among children. Dev Sci.

[R43] Lee S, Lang A (2015). Redefining media content and structure in terms of available resources: toward a dynamic human-centric theory of communication. Communic Res.

[R44] Li H, Subrahmanyam K, Bai X, Xie X, Liu T (2018). Viewing fantastical events versus touching fantastical events: short-term effects on children’s inhibitory control. Child Dev.

[R45] Lillard AS, Peterson J (2011). The immediate impact of different types of television on young children’s executive function. Pediatrics.

[R46] Lui KY, Hendry A, Fiske A, Dvergsdal H, Holmboe K (2021). Associations between touchscreen exposure and hot and cool inhibitory control in 10-month-old infants. Infant Behav Dev.

[R47] McHarg G, Ribner AD, Devine RT, Hughes C, New FAMS Study Team (2020). Infant screen exposure links to toddlers’ inhibition, but not other EF constructs: a propensity score study. Infancy.

[R48] McNeill J, Howard SJ, Vella SA, Cliff DP (2019). Longitudinal associations of electronic application use and media program viewing with cognitive and psychosocial development in preschoolers. Acad Pediatr.

[R49] Miller CJ, Marks DJ, Miller SR, Berwid OG, Kera EC, Santra A (2007). Brief report: Television viewing and risk for attention problems in preschool children. J Pediatr Psychol.

[R50] Murphy K, Shin M (2022). Frequent media multitasking is not associated with better cognitive flexibility. J Cognit Psychol.

[R51] Nathanson AI, Aladé F, Sharp ML, Rasmussen EE, Christy K (2014). The relation between television exposure and executive function among preschoolers. Dev Psychol.

[R52] Neumann MM, Neumann DL (2014). Touch screen tablets and emergent literacy. Early Childh Educ J.

[R53] Portugal AM, Hendry A, Smith TJ, Bedford R (2023). Do pre-schoolers with high touchscreen use show executive function differences?. Comput Human Behav.

[R54] Power J, van IJzendoorn M, Lewis AJ, Chen W, Galbally M (2021). Maternal perinatal depression and child executive function: a systematic review and meta-analysis. J Affect Disord.

[R55] Rhodes SM, Stewart TM, Kanevski M (2020). Immediate impact of fantastical television content on children’s executive functions. Br J Dev Psychol.

[R56] Ribner A, Fitzpatrick C, Blair C (2017). Family socioeconomic status moderates associations between television viewing and school readiness skills. J Dev Behav Pediat.

[R57] Scionti N, Marzocchi GM (2021). The dimensionality of early executive functions in young preschoolers: comparing unidimensional versus bidimensional models and their ecological validity. Child Neuropsychol.

[R58] Senn TE, Espy KA, Kaufmann M (2004). Using path analysis to understand executive function organization in preschool children. Dev Neuropsychol.

[R59] Subrahmanyam K, Greenfield PM (2008). Virtual worlds in development: implications of social networking sites. J Appl Dev Psychol.

[R60] Taherian Kalati A, Kim MS (2022). What is the effect of touchscreen technology on young children’s learning?: a systematic review. Educ Inform Technol.

[R61] Toplak ME, West RF, Stanovich KE (2013). Practitioner review: do performance-based measures and ratings of executive function assess the same construct?. J Child Psychol Psychiat.

[R62] Wiebe SA, Espy KA, Charak D (2008). Using confirmatory factor analysis to understand executive control in preschool children: I. Latent structure. Dev Psychol.

[R63] Zelazo D, Carlson SM (2012). Hot and cool executive function in childhood and adolescence: development and plasticity. Child Dev Perspect.

