# Supplementary material for: Concurrent and longitudinal associations between touchscreen use and executive functions at preschool-age
Source: Front Dev Psychol. Author manuscript; Available in PMC 2025 Aug 18. (PMC7618025; doi:10.3389/fdpys.2024.1422635)
Supplement: Supplementary material [file EMS207788-supplement-Supplementary_material.PDF]

Supplementary Materials

Supplementary Materials 1: Touchscreen Use Questionnaire (TUQ)

Touchscreen use

Touchscreen Questionnaire

How often does your child do these things with a smartphone, tablet or other touchscreen device?

	Never	Once per month or less	2-4 times per month	5-8 times per month	More than twice per week	2-4 times per week	Most days
Watch videos or look at photos without touching the screen (i.e., with an adult controlling the device)	○	○	○	○	○	○	○
Scroll/swipe through photos or videos	○	○	○	○	○	○	○
Have video calls with a loved one	○	○	○	○	○	○	○
Play simple games (e.g., tapping or swiping a cartoon)	○	○	○	○	○	○	○
Do drawings or scribbles	○	○	○	○	○	○	○

How much does your child enjoy playing on a touchscreen device (this could involve playing games or just tapping/swiping through photos and videos)?

Does not have the opportunity	Not at all	A little	A moderate amount	A lot	A great deal
○	○	○	○	○	○

In the past week, roughly how long in total did your child spend **looking at (but not touching)** a touchscreen device? *(Not including visits to the BabyLab)*

Less than 5 minutes	5-20 minutes	20-60 minutes	1-2 hours	2-4 hours	4-6 hours	7 or more hours
○	○	○	○	○	○	○

In the past week, roughly how long in total did your child spend **interacting with (tapping or swiping)** a touchscreen device? *(Not including visits to the BabyLab)*

Less than 5 minutes	5-20 minutes	20-60 minutes	1-2 hours	2-4 hours	4-6 hours	7 or more hours
○	○	○	○	○	○	○

At what age did your child first:

	Before 6 months	6-9 months	9-12 months	12-15 months	15-18 months	18-21 months	21-24 months	Has not done this yet
Watch videos on a phone or tablet	○	○	○	○	○	○	○	○
Scroll/swipe through photos or videos	○	○	○	○	○	○	○	○
Have video calls with a loved one	○	○	○	○	○	○	○	○
Play simple games (e.g tapping or swiping a cartoon)	○	○	○	○	○	○	○	○
Do drawings or scribbles	○	○	○	○	○	○	○	○

Supplementary Materials 2: Correlational Analysis Assumption Checks

Normal Distribution: Kolmogorov-Smirnov test of normality

If the *p*-value is less than the significance level (typically 0.05), then the null hypothesis is rejected, and it is concluded that the variable's values are not normally distributed. These *p*-values which suggest the data is skewed have an asterisk (*) in the table below.

Kolmogorov-Smirnov test of normality for each variable

**Variable**	**Statistic**	**df**	***p value***
Maternal Education	.11	101	.01*
42-month Passive Touchscreen Use	.20	101	<.001*
42-month Active Touchscreen Use	.20	101	<.001*
Average Passive Touchscreen Use	.13	101	<.001*
Average Active Touchscreen Use	.15	101	<.001*
ISCI Scores	.10	101	.02*
FI Scores	.10	101	.01*
EMI Scores	.08	101	.07

Note. ISCI = Inhibitory Self-Control Index; FI = Flexibility Index; Emergent Metacognition Index

Normal Distribution: histograms and normal Q-Q plots

For a normal distribution, each histogram should look symmetric around the mean of the distribution. In the Quantile-Quantile (Q-Q) plots, the data are plotted against a theoretical normal distribution in such a way that the points should form an approximate straight line.

Histogram of Maternal Years in Education

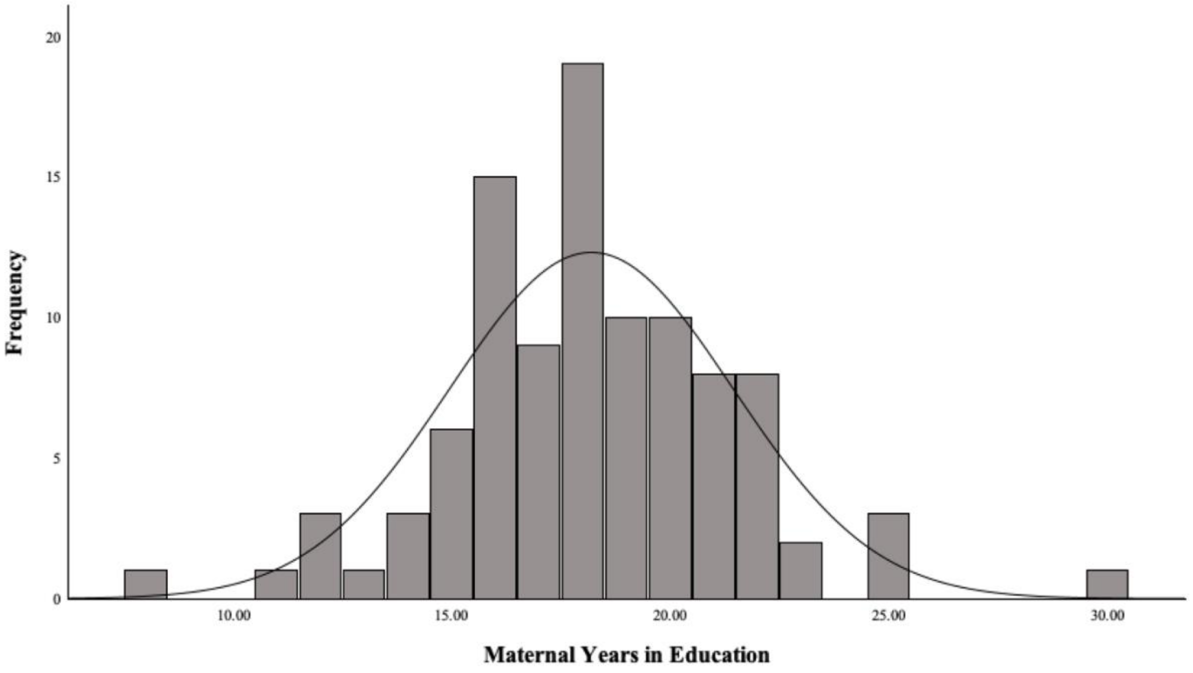

Normal Q-Q Plot of Maternal Years in Education

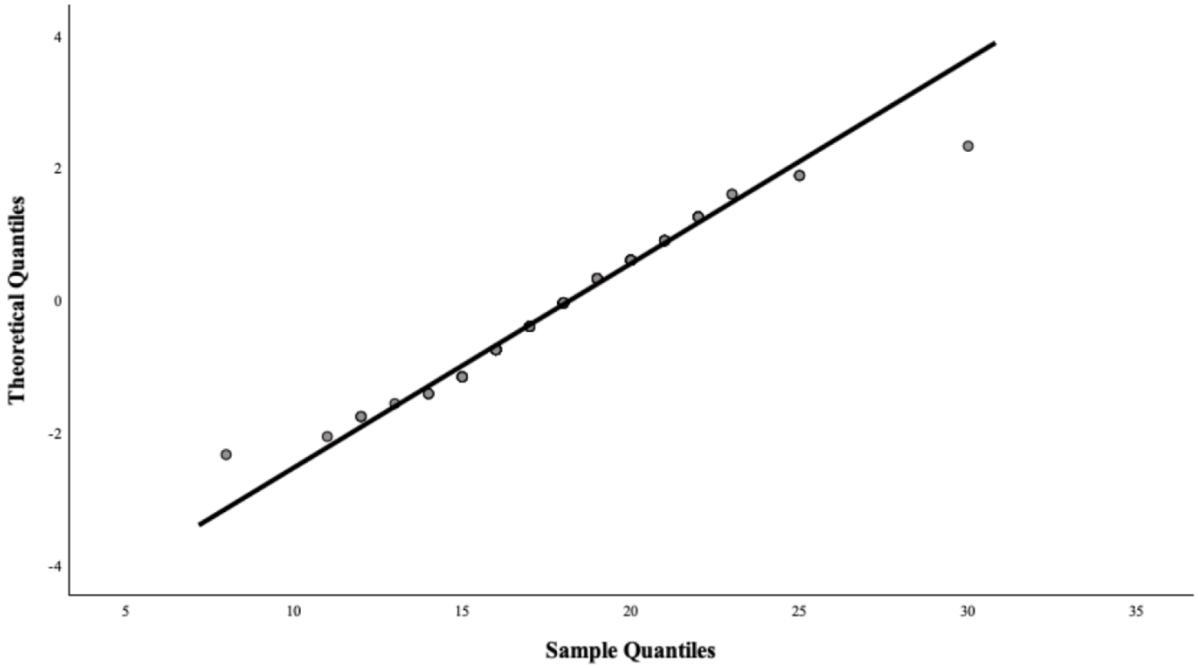

Histogram of 42-month Passive Touchscreen Use

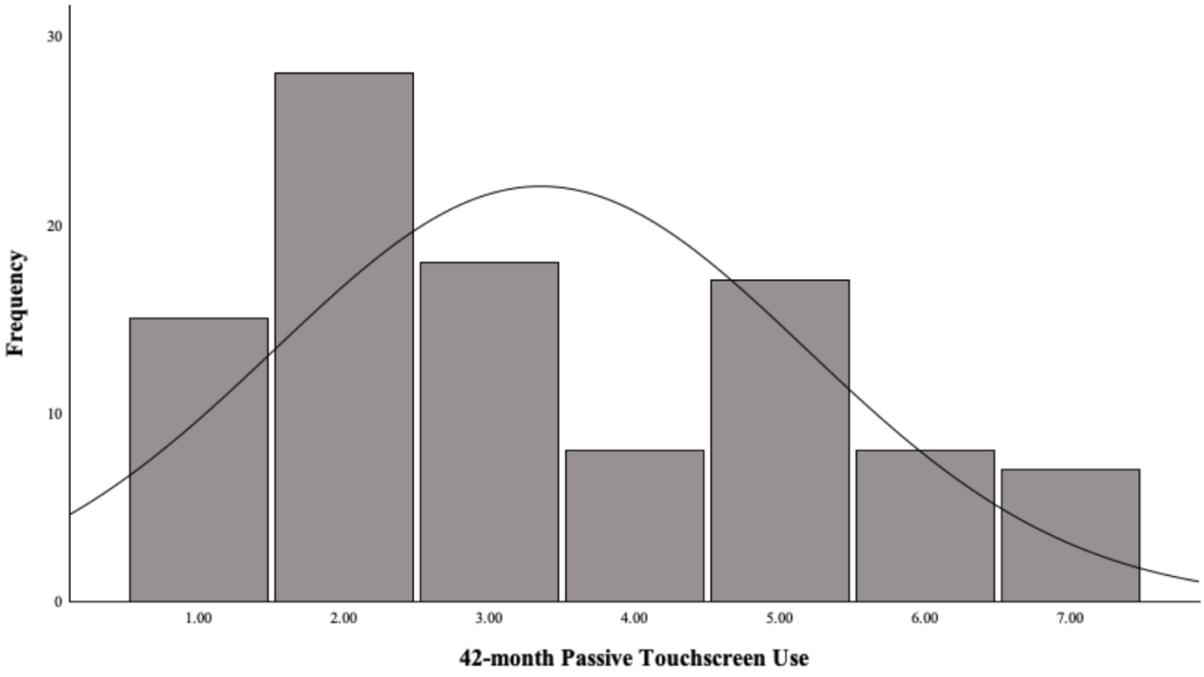

Normal Q-Q Plot of 42-month Passive Touchscreen Use

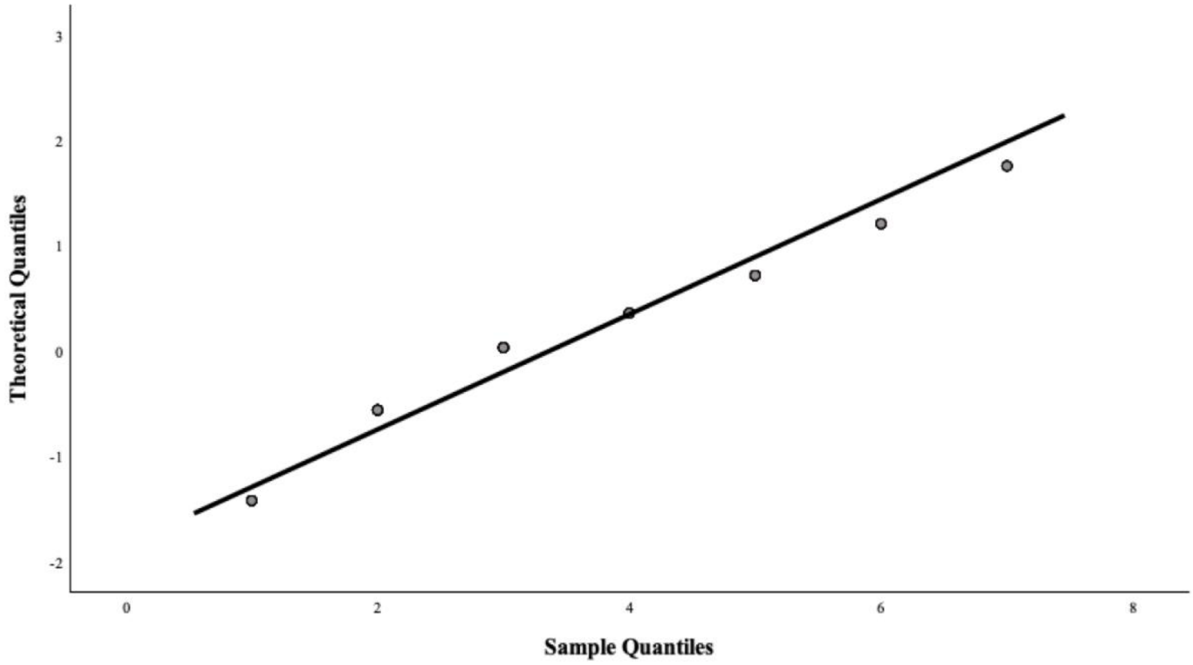

Histogram of 42-month Active Touchscreen Use

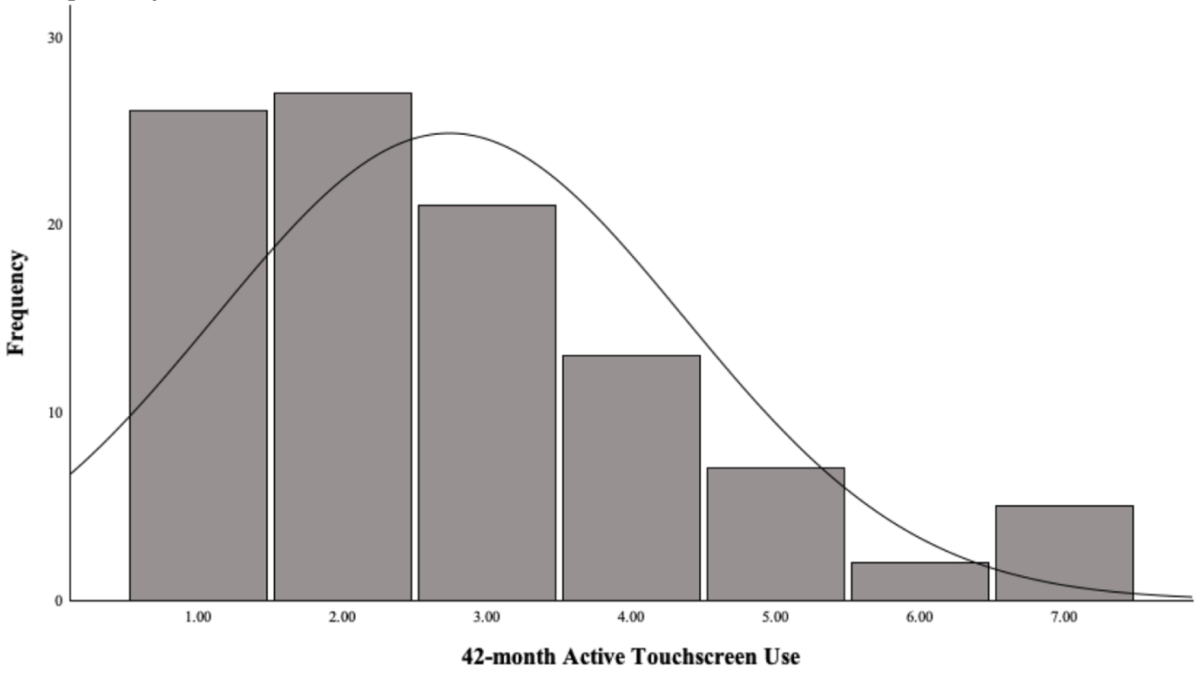

Normal Q-Q Plot of 42-month Active Touchscreen Use

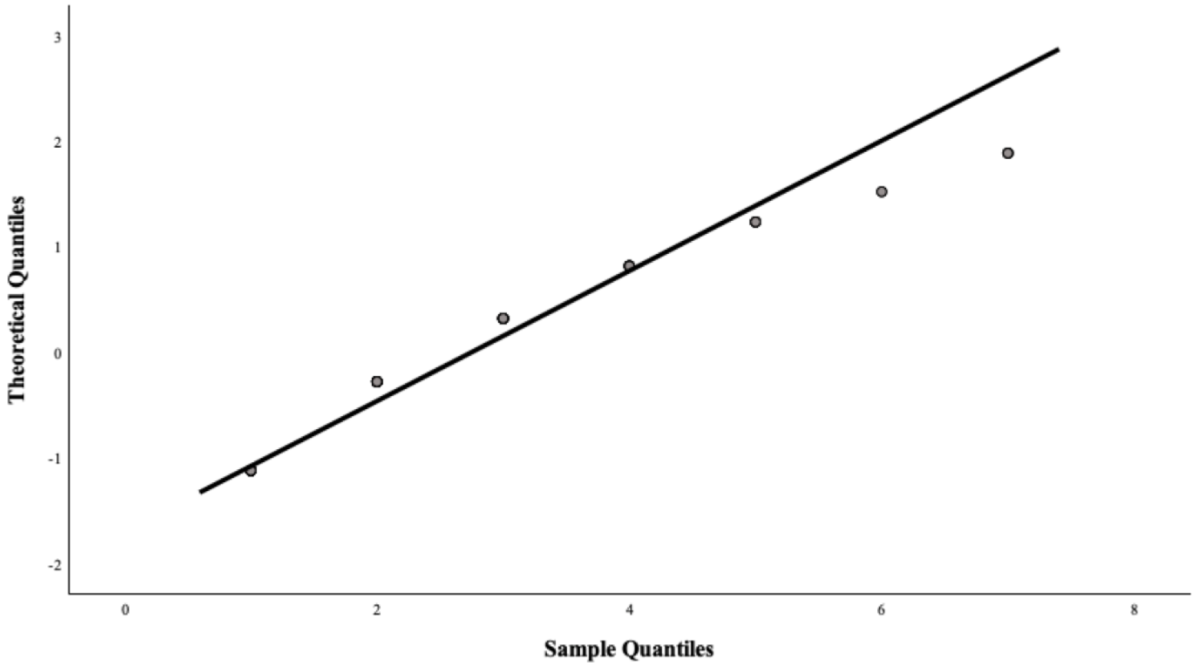

Histogram of Average Passive Touchscreen Use

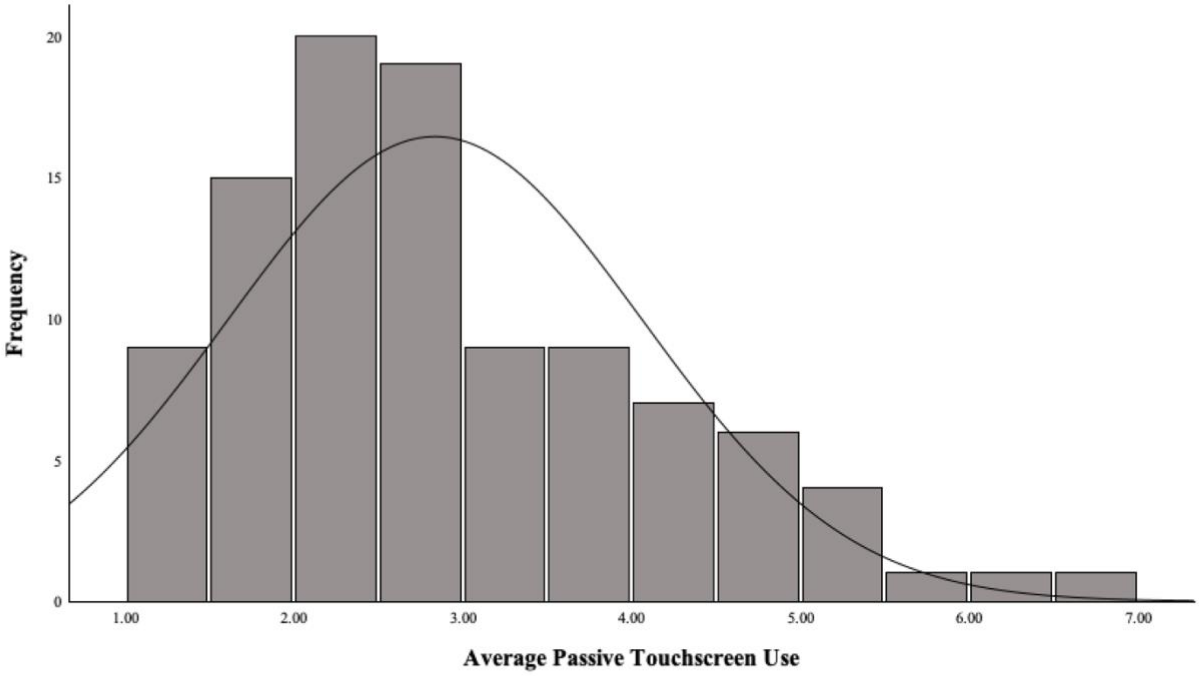

Normal Q-Q Plot of Average Passive Touchscreen Use

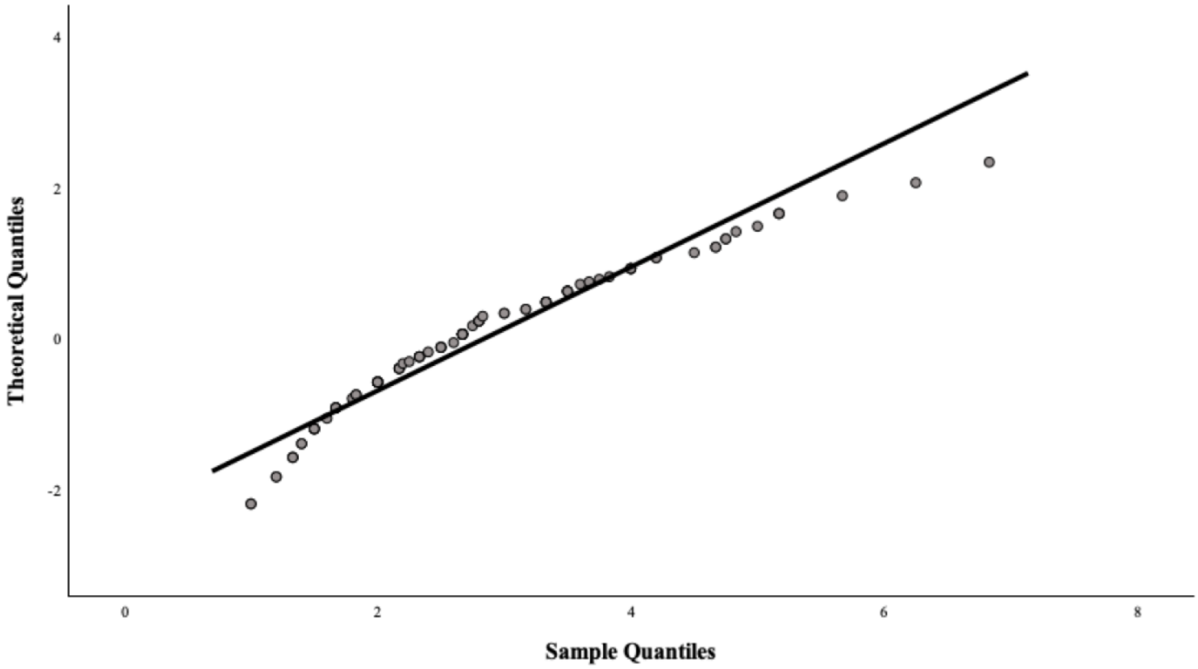

Histogram of Average Active Touchscreen Use

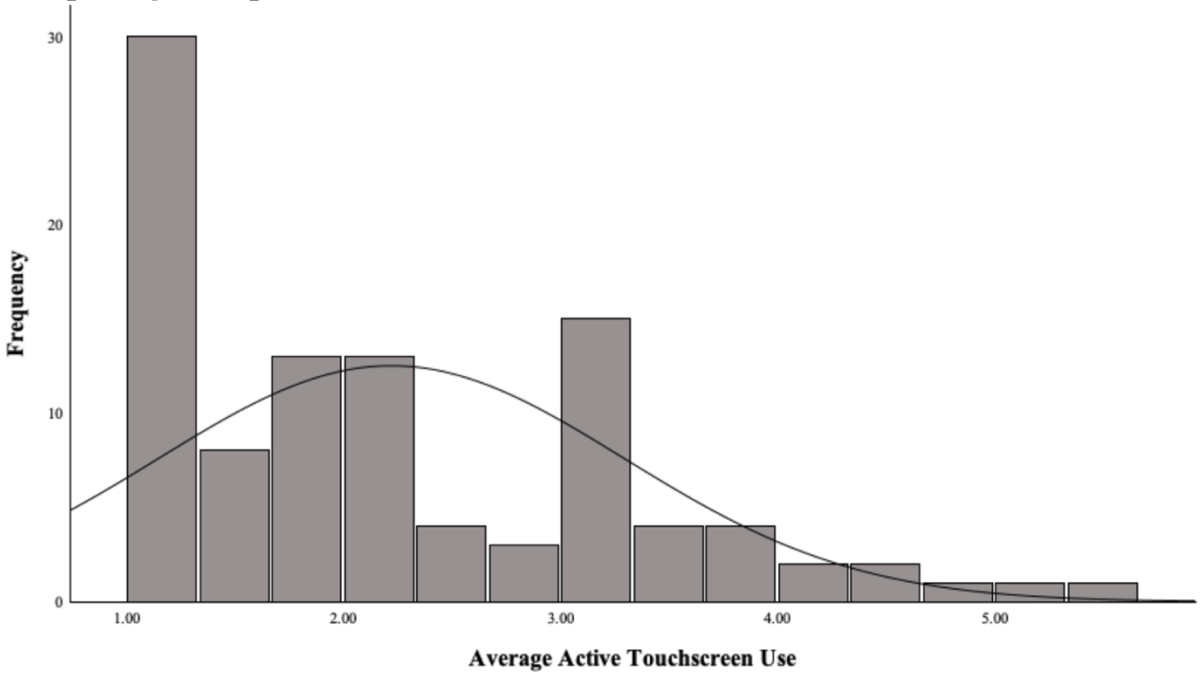

Normal Q-Q Plot of Average Active Touchscreen Use

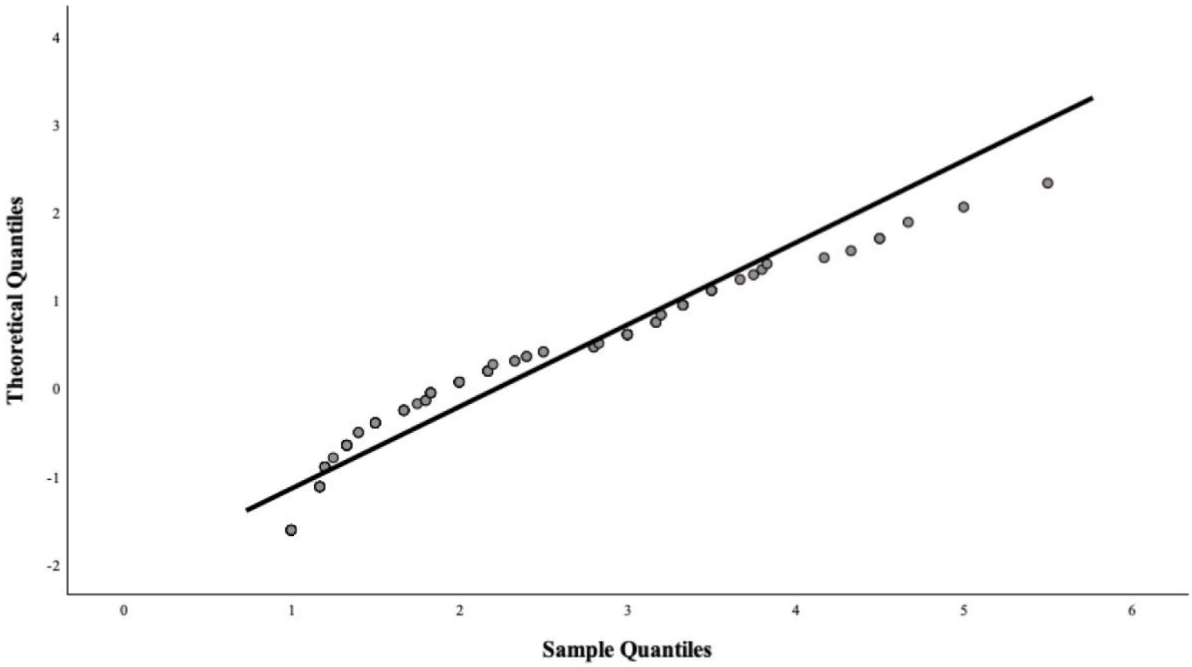

Histogram of Inhibitory Self-Control Index Scores

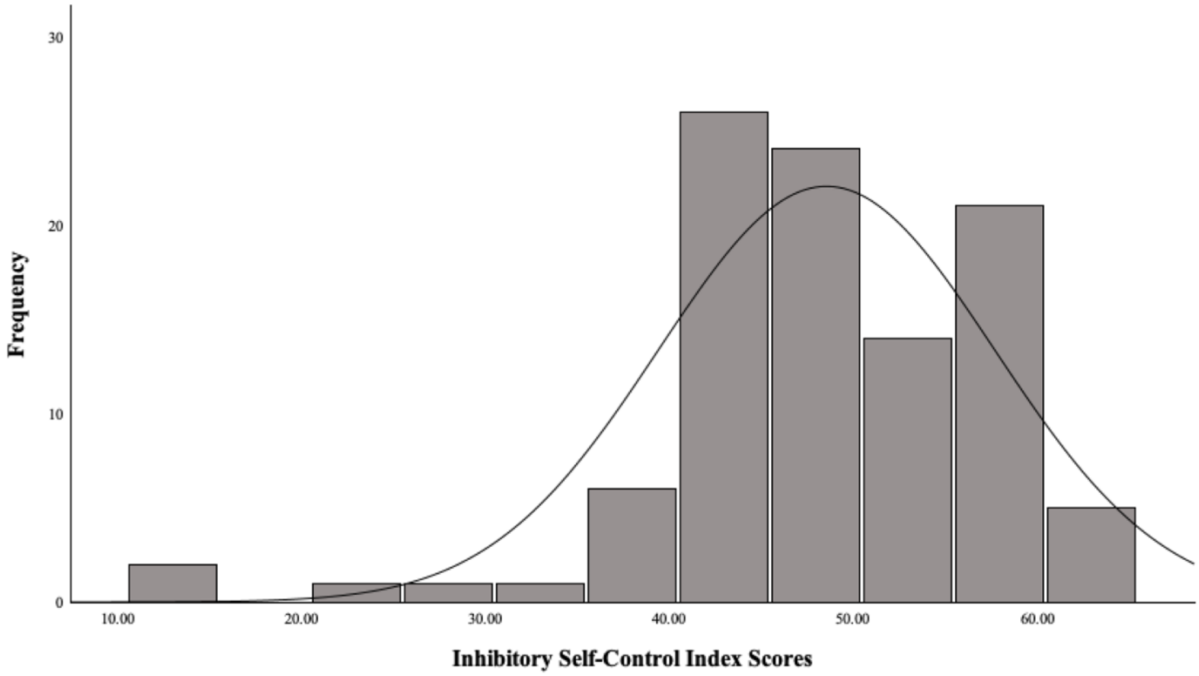

Normal Q-Q Plot of Inhibitory Self-Control Index Scores

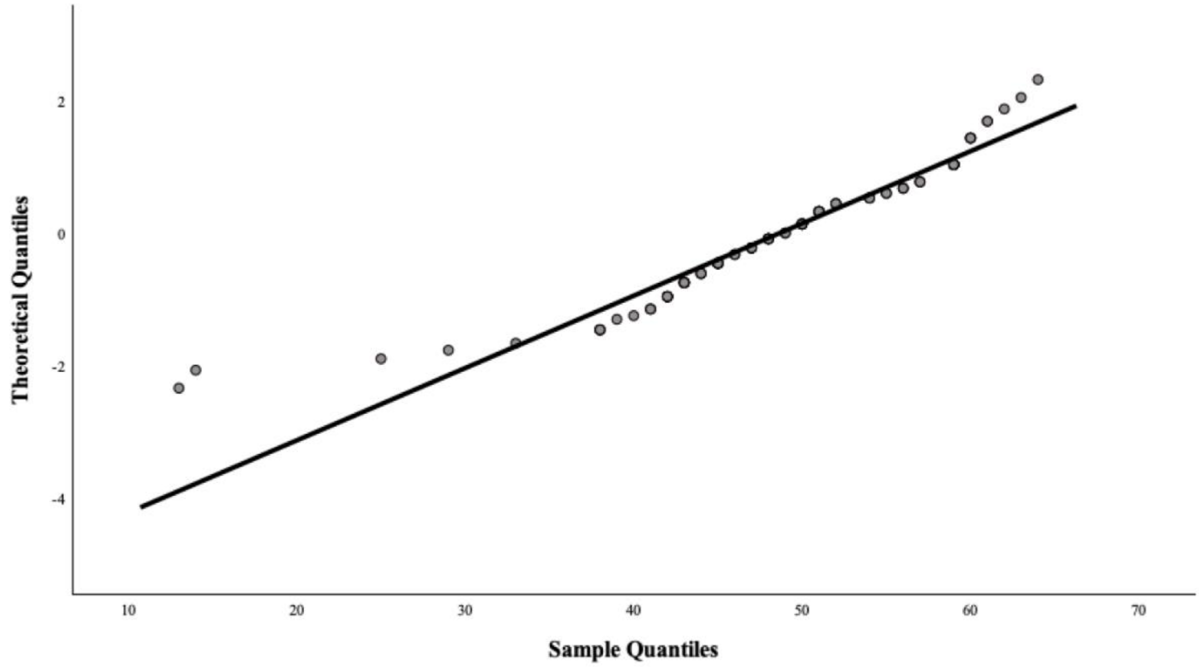

Histogram of Flexibility Index Scores

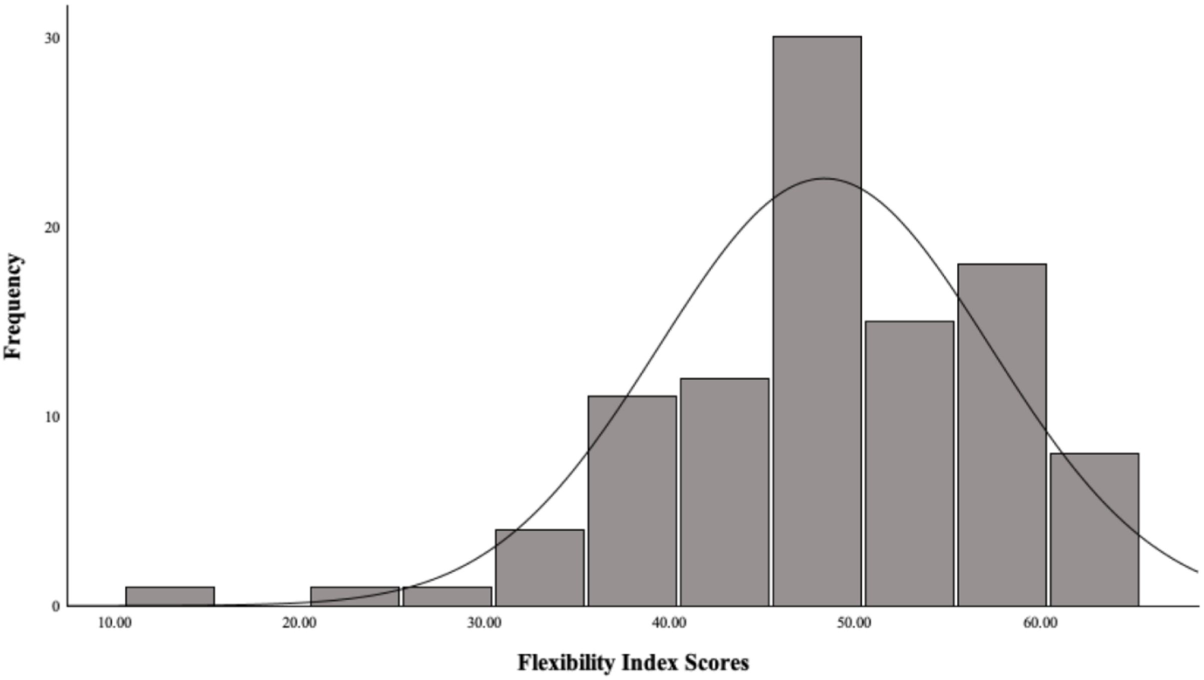

Normal Q-Q Plot of Flexibility Index Scores

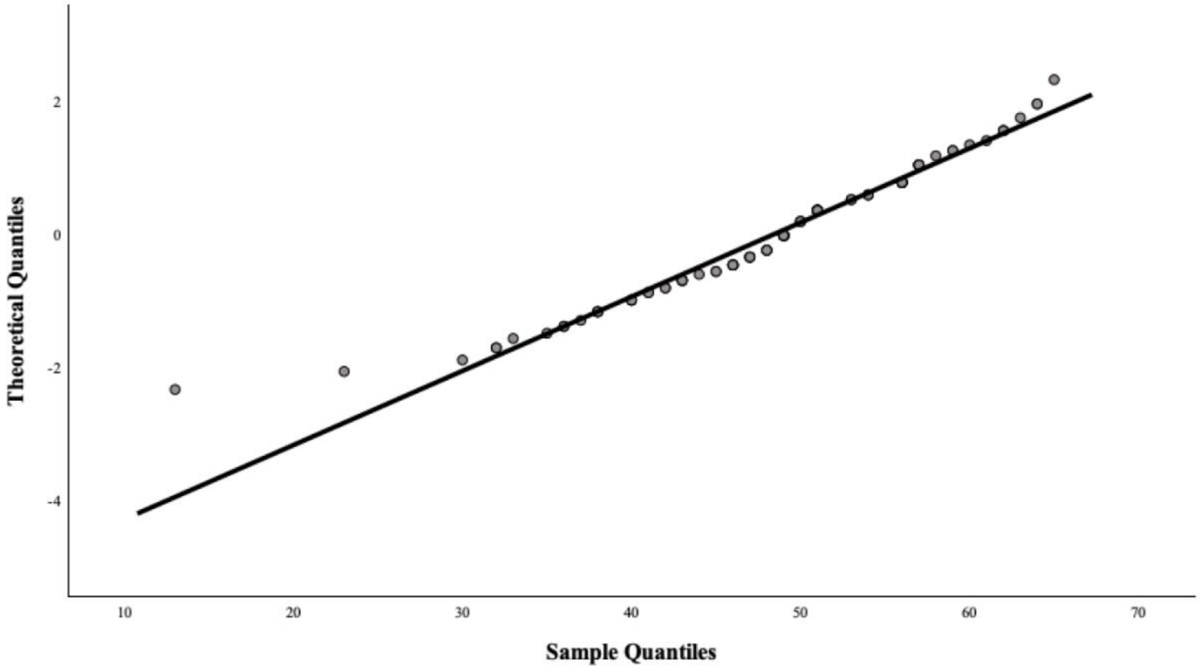

Histogram of Emergent Metacognition Index Scores

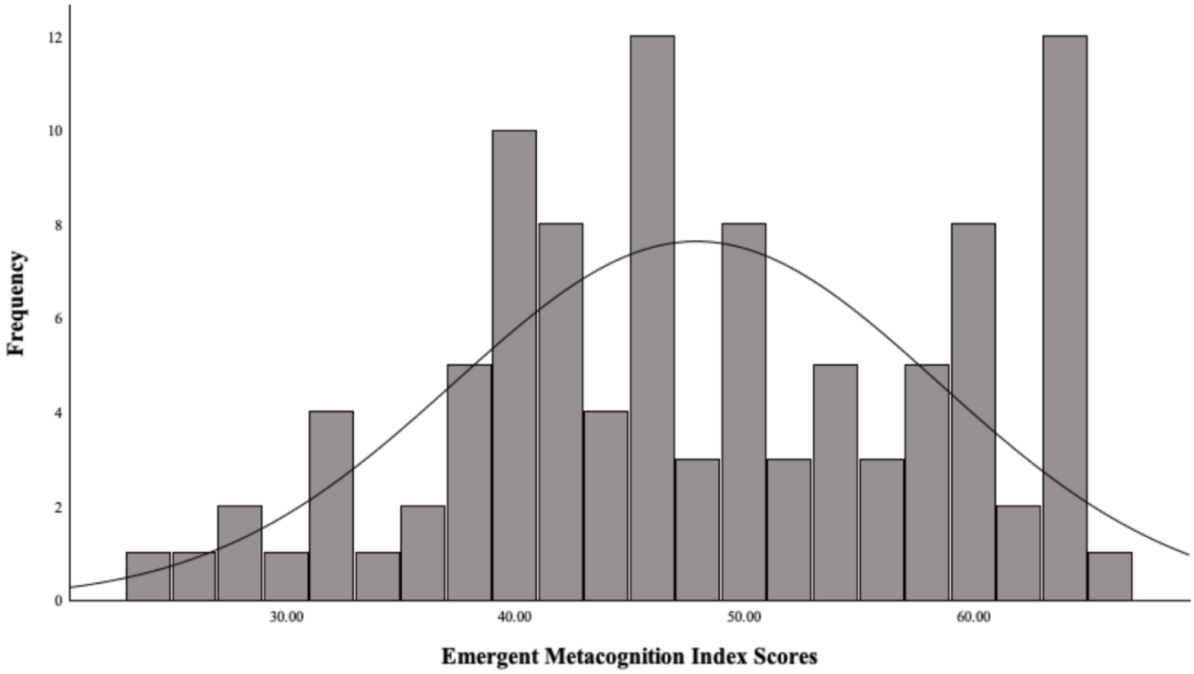

Normal Q-Q Plot of Emergent Metacognition Index Scores

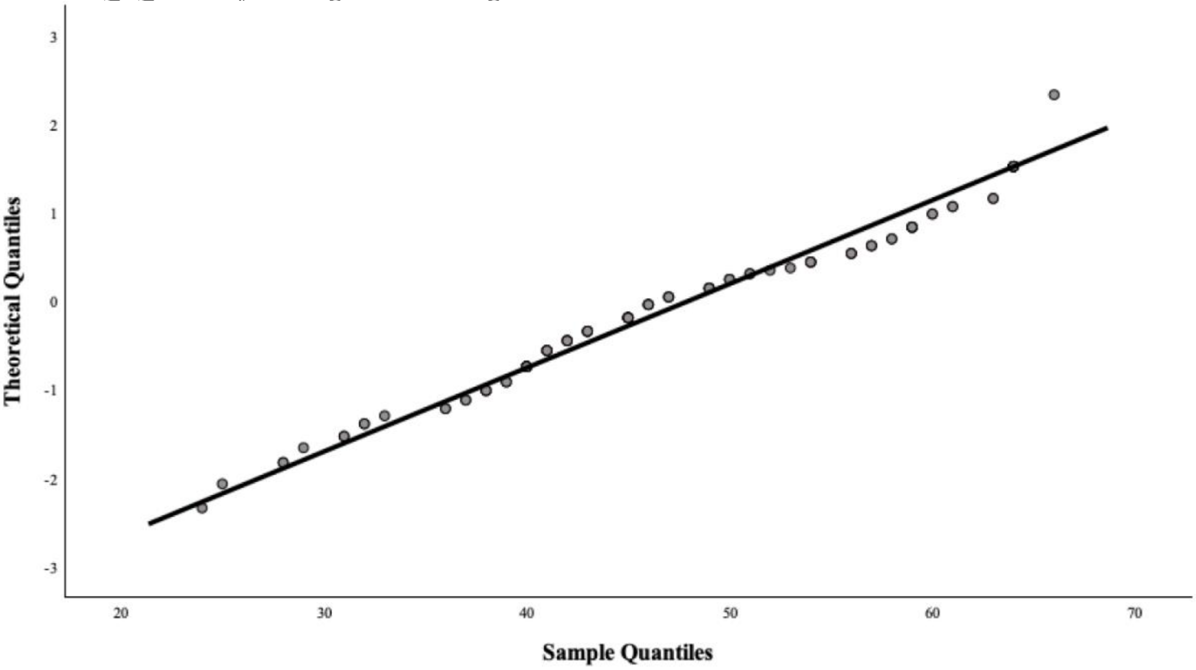

Supplementary Materials 3: Multivariate Multiple Regression Assumption Checks

Normal Distribution: histogram of standardised residuals and normality P-P plots of regression standardised residuals

In the normality P-P plots of regression standardised residuals, the points should be in a reasonably straight line from bottom left to top right. The histograms of standardised residuals should have a roughly symmetrical bell-shape, with a single peak in the middle of the distribution.

<u>Inhibitory Self-Control Index Scores</u>

Histogram of standardised residuals for the BRIEF-P Inhibitory Self-Control Index Scores

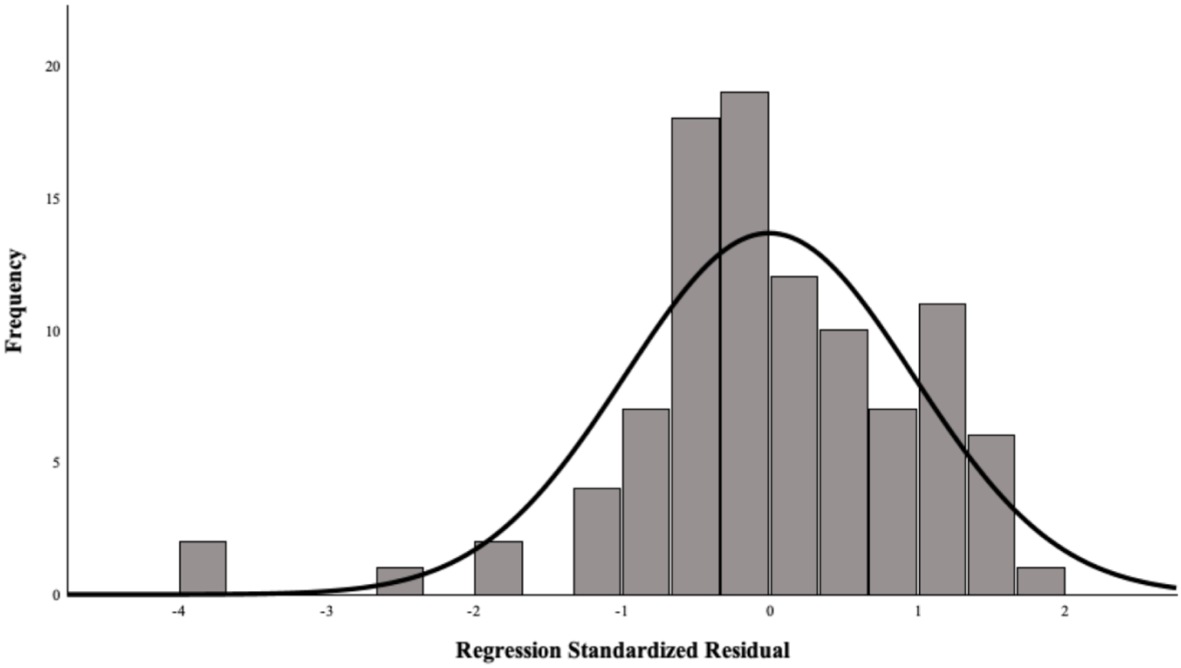

Normality P-P plots of standardised residuals for the BRIEF-P Inhibitory Self-Control Index Scores

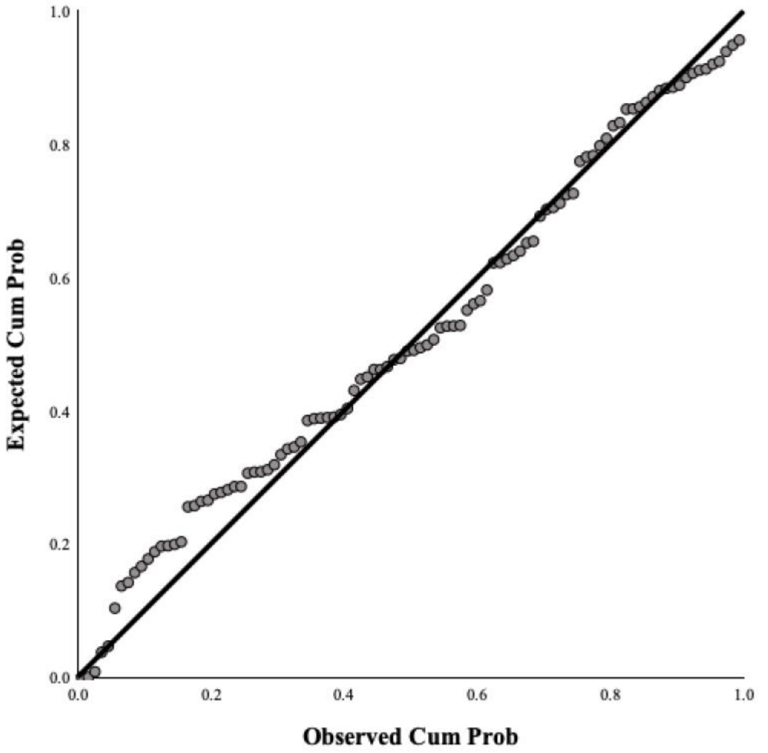

<u>Flexibility Index Scores</u>

Histogram of standardised residuals for the BRIEF-P Flexibility Index Scores

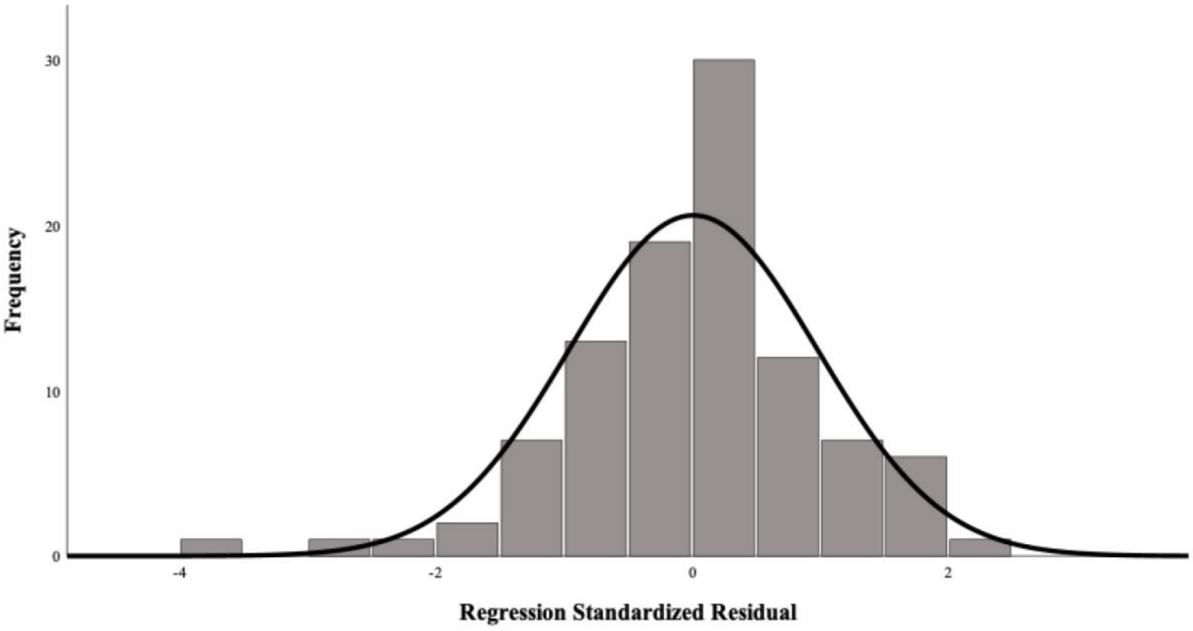

Normality P-P plots of standardised residuals for the BRIEF-P Flexibility Index Scores

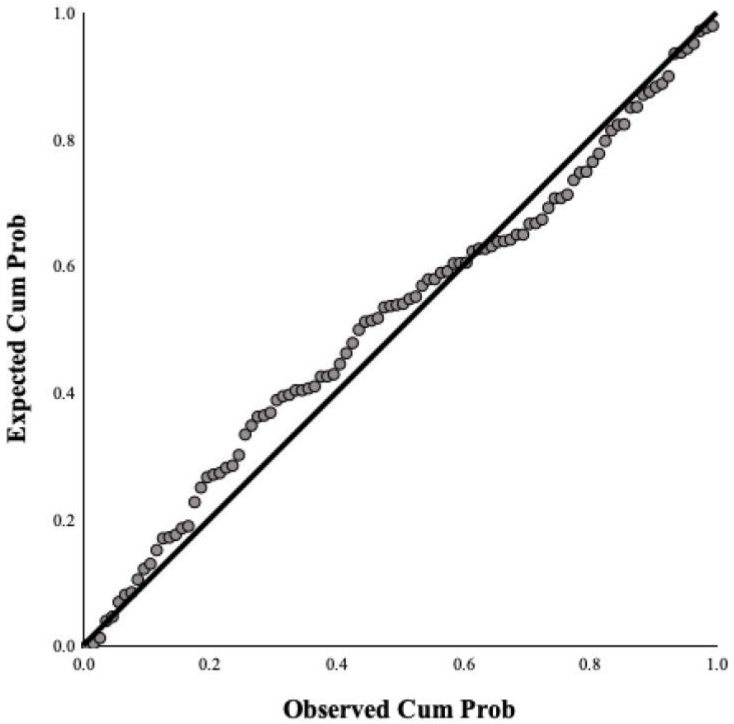

Histogram of standardised residuals for the BRIEF-P Emergent Metacognition Index Scores

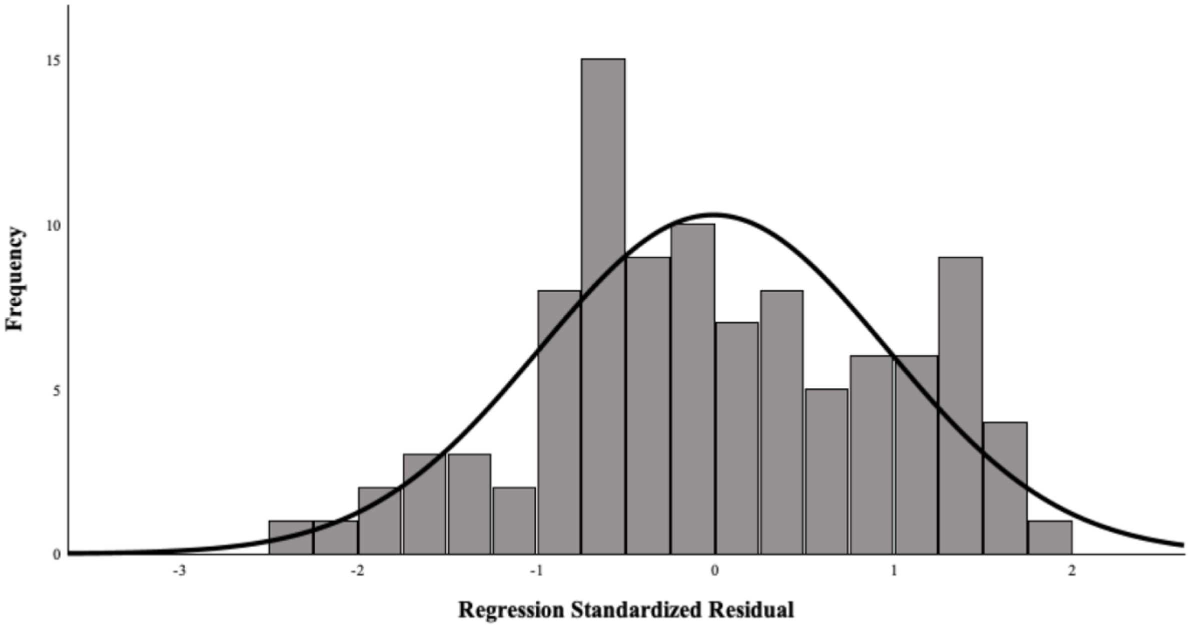

Normality P-P plots of standardised residuals for the BRIEF-P Emergent Metacognition Index Scores

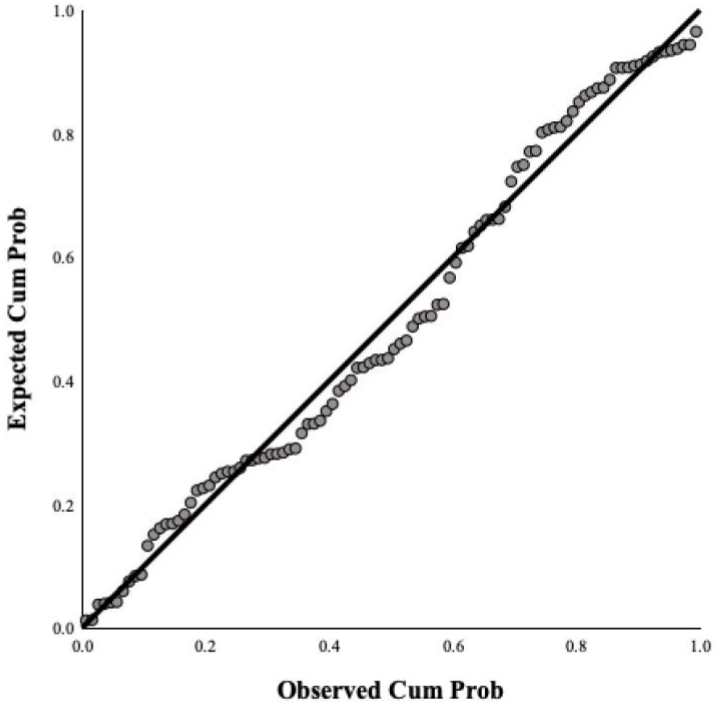

Normal Distribution: Cook's distances

Mahalanobis distances should all be < 22.458 (critical chi-square value for df = 6; Tabachnick and Fidell, 2019). Cook's Distance should be < 1 (Tabachnick and Fidell, 2013).

Table of Cook's distances

Variable	Min	Max	Mean	SD	N
ISCI	<.001	.22	.01	.03	100
FI	<.001	.23	.01	.03	100
EMI	<.001	.09	.01	.02	100

Note. ISCI = Inhibitory Self-Control Index; FI = Flexibility Index; Emergent Metacognition Index.

Table of Mahalanobis distances

Variable	Min	Max	Mean	SD	N
ISCI	1.72	21.25	5.94	3.68	100
FI	<.001	21.25	5.94	3.68	100
EMI	<.001	21.25	5.94	3.68	100

Note. ISCI = Inhibitory Self-Control Index; FI = Flexibility Index; Emergent Metacognition Index.

Homoscedasticity: scatterplots of standardised and predicted residuals
The points should be equally dispersed around the regression line in a cloud-like pattern to suggest variance in the residuals is equal.

<u>Inhibitory Self-Control Index Scores</u>
Scatterplot of standardised and predicted residuals for the BRIEF-P Inhibitory Self-Control Index Scores

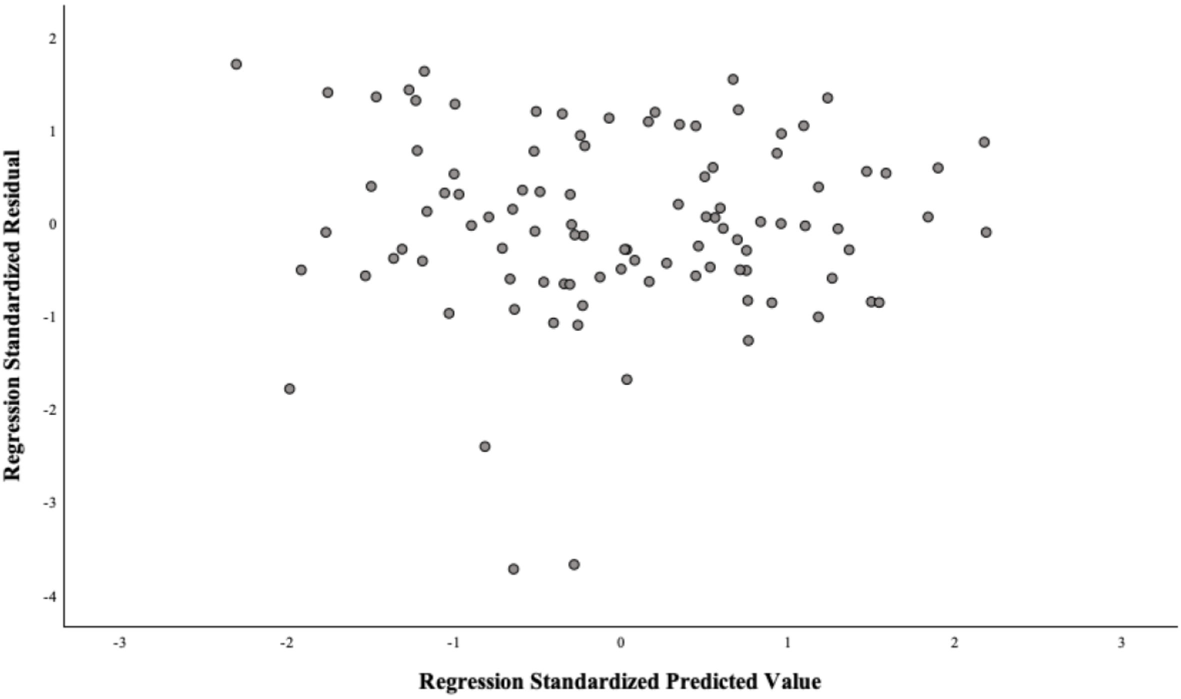

Scatterplot of standardised and predicted residuals for the BRIEF-P Flexibility Index Scores

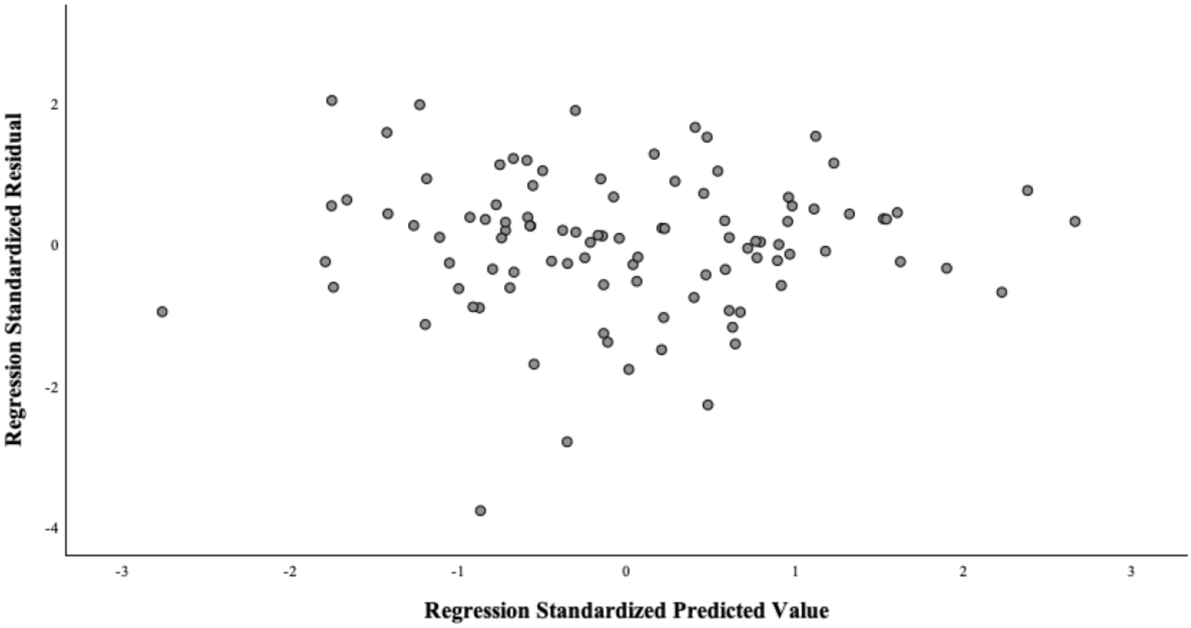

Scatterplot of standardised and predicted residuals for the BRIEF-P Emergent Metacognition Index Scores

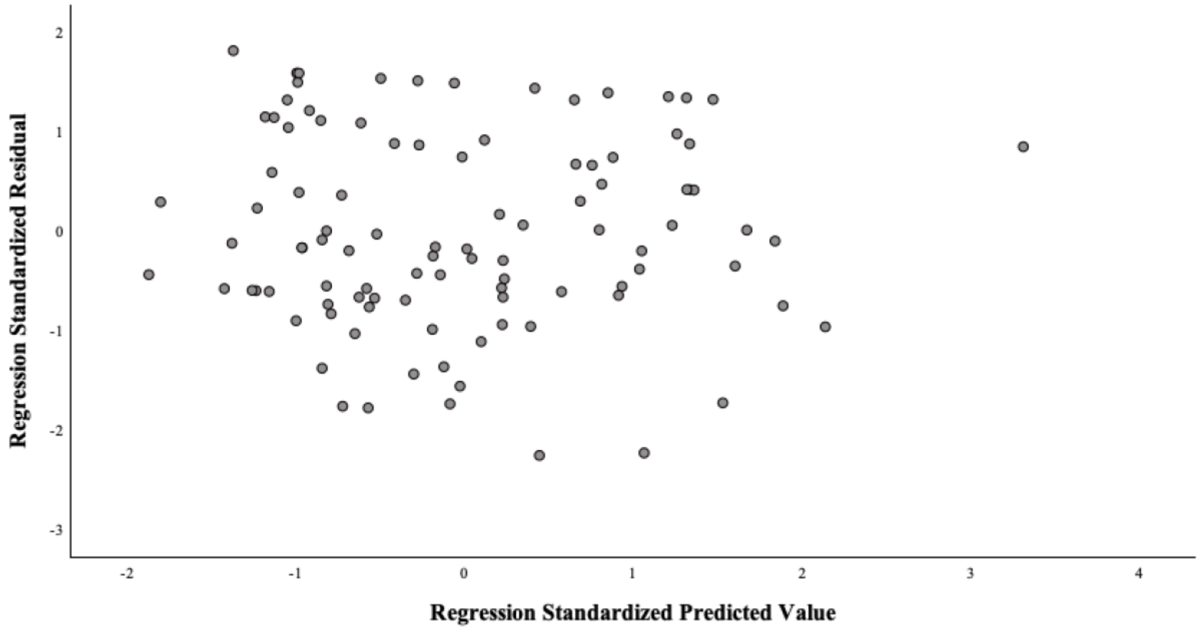

Multicollinearity: tolerance and VIF statistics

Tolerance values should be .10 <, and maximum VIF scores should all be between 1-10 to suggest that two or more independent variables are not highly correlated (Daoud, 2017; Pallant, 2020).

Table of Minimum Tolerance and Maximum VIF Scores for Each Independent Variable

Collinearity Statistics	Tolerance	VIF
Child Sex	.91	1.10
Maternal Education	.81	1.23
42-month Passive Touchscreen Use	.36	2.78
42-month Active Touchscreen Use	.38	2.65
Average Passive Touchscreen Use	.35	2.84
Average Active Touchscreen Use	.30	3.34

References

Daoud, J.I., 2017, December. Multicollinearity and regression analysis. In *Journal of Physics: Conference Series* (Vol. 949, No. 1, p. 012009). IOP Publishing. https://doi.org/10.1088/1742-6596/949/1/012009

Tabachnick, B. G., & Fidell, L. S. (2013). Using multivariate statistics (6th ed). Boston: Pearson Education.

Tabachnick, B. G., & Fidell, L. S. (2013). Using multivariate statistics (7th ed). Boston: Pearson Education.

Supplementary Materials 4: Correlations Between the Independent Variables

Variables	1	2	3	4	5	6
1. Child Sex	—					
2. Maternal Years in Education	-.09	—				
3. 42-month Passive Touchscreen Use	.20	.29*	—			
4. 42-month Active Touchscreen Use	.19	.003	.35*	—		
5. Average Passive Touchscreen Use	.21	.22*	.77*	.35*	—	
6. Average Active Touchscreen Use	.22	-.08	.48*	.80*	.53*	—

*Significant Benjamini-Hochberg adjusted *p*-value corrected to the alpha level of .05 for the false discovery rate (15 family-wise comparisons), two-tailed

Supplementary Materials 5: Frequency of Touchscreen Use from 10-to-42-months
In addition to reporting the duration of time their child spent *looking at* (but not touching) a touchscreen device in the past week, and the duration their child spent *interacting with* (tapping or swiping) a touchscreen device in the past week (on a 7-point Likert scale from 'Less than 5 minutes' to '7 or more hours'), parents also rated how frequently their child did the following actions on a touchscreen on a 7-point Likert scale (1 = *Never*, 7 = *Most days*): (a) watch videos or look at photos without touching the screen, (b) scroll/swipe through photos or videos, (c) have video calls with loved ones, (d) play simple games, and (e) do drawings/scribbles.

The touchscreen frequency items related to *active* touchscreen use were averaged together to form a frequency of active touchscreen use scale (i.e., frequency of scrolling/swiping through photos or videos; frequency of playing simple games; frequency of doing drawings/scribbles). The having 'video calls with loved ones' item was removed from the active touchscreen use frequency scale because screen time guidelines tend to exclude video calling from their recommended restrictions (Pappas, 2020). The single touchscreen use item related to *passive* touchscreen use (i.e., frequency of watching videos or looking at photos) was used as a measure of frequency of passive touchscreen use. The figure below shows the mean frequency of passive and active touchscreen use from 10-to-42-months-old.

Mean Passive and Active Frequency of Touchscreen use at each of the six timepoints from 10-to-42-months-old

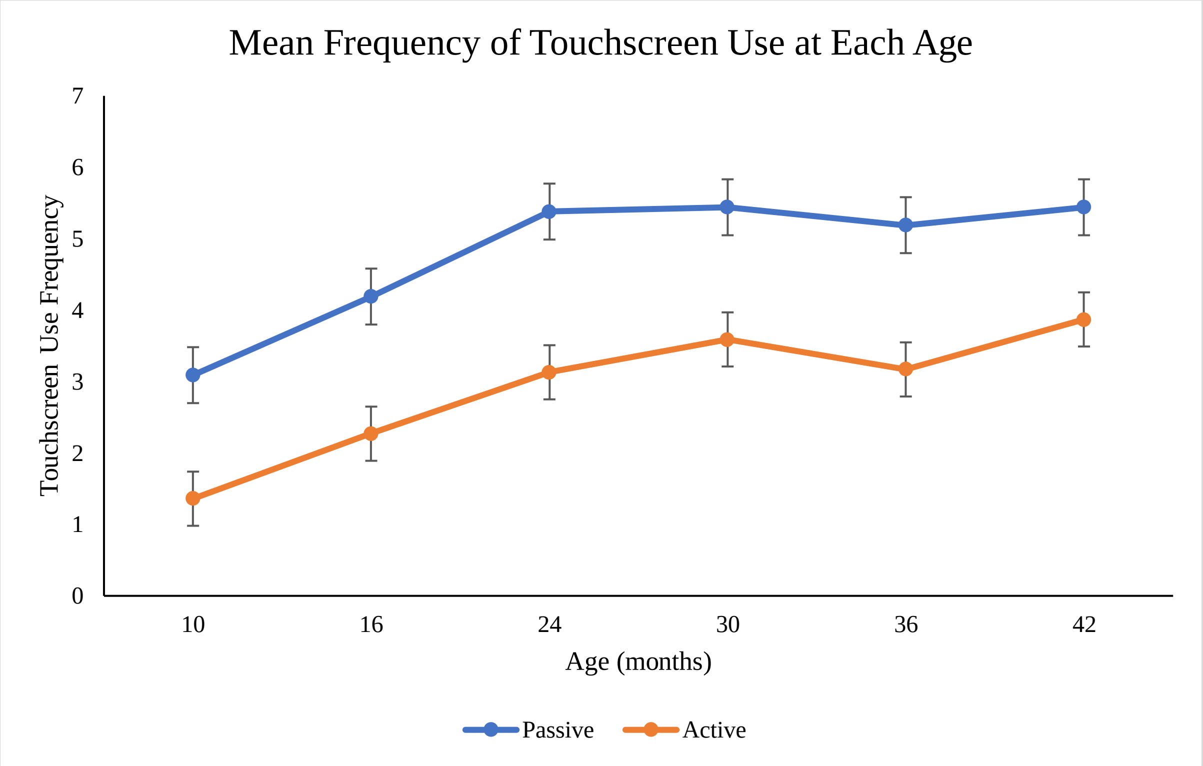

Calculating Passive and Active Touchscreen Frequency Scores
Because data were collected longitudinally across six timepoints, participants' average frequencies of passive and active touchscreen use over time were calculated for each child. Scores of frequency of passive touchscreen use (from the 'frequency of watching videos or looking at photos' item) from 10-to-42-months of age were averaged to calculate an average frequency of passive touchscreen use score across the first 3.5-years of life. Scores of

frequency of active touchscreen use (from the frequency of ‘scrolling/swiping through photos or videos’ item, the frequency of ‘playing simple games’ item, and the frequency of ‘doing drawings/scribbles’ item) from 10-to-42-months of age were averaged to calculate an average frequency of active touchscreen use score across the first 3.5-years of life. These average frequency of passive touchscreen use and average frequency of active touchscreen use scores had good internal consistency across timepoints (Cronbach's = .73; Cronbach's = .87, respectively). The table below shows descriptive statistics for the sample's frequency of passive and active touchscreen use at each of the six timepoints from 10-to-42-months of age.

Descriptive Statistics of Frequency of Passive and Active Touchscreen Use at Each Timepoint

	**10-months**	**16-months**	**24-months**	**30-months**	**36-months**	**42-months**	**Average (10-42 months)**
**Frequency of Passive Use**							
Mean	3.09	4.19	5.38	5.44	5.19	5.44	4.73
SD	1.96	2.14	1.55	1.66	1.61	1.36	1.13
**Frequency of Active Use**							
Mean	1.36	2.27	3.13	3.59	3.17	3.87	2.85
SD	0.61	1.33	1.71	1.80	1.66	1.61	1.09

Correlations between Frequency of Touchscreen Use and Executive Functions

Whilst touchscreen use is typically measured by duration of usage time (e.g., in a week), a correlational analysis between frequency of touchscreen use and EF skills (as measured by the BRIEF-P) was also run. This was to investigate whether duration and frequency of touchscreen use have different associations with preschool EF skills. As seen in Table 6, no significant correlations were found between any of the frequency of touchscreen use measures and EF skills (both before and after correcting for multiple comparisons using the Benjamini-Hochberg procedure). These null results differ to a significant positive correlation between duration of 42-month active touchscreen use and scores on the BRIEF-P Flexibility Index (r_S = .27, p = .01, but this did not survive correction for multiple comparisons; Benjamini-Hochberg adjusted p-value = .08). This may suggest that frequency and duration may be two different aspects of touchscreen use.

References

Pappas, S. (2020). What do we really know about kids and screens? *Monitor on Psychology, 51*(3), 42. https://www.apa.org/monitor/2020/04/cover-kids-screens
